# Supplementary material for: The SPOC domain is a phosphoserine binding module that bridges transcription machinery with co- and post-transcriptional regulators
Source: Nat Commun. 2023 Jan 11;14:166. doi: 10.1038/s41467-023-35853-1 (PMC9834408; doi:10.1038/s41467-023-35853-1)
Supplement: Supplementary file 1 — Supplementary Information [file 41467_2023_35853_MOESM1_ESM.pdf]

Supplementary Table 1. X-ray data collection and refinement

	**SHARP SPOC:1xS5P CTD**	**RBM15 SPOC**
Source	MASSIF1 (ESRF, Grenoble, France)	I04 (DLS, Didcot, UK)
Wavelength (Å)	0.9660	0.9795
Resolution (Å)	36.58-1.55 (1.58-1.55)	57.45-1.45 (1.47-1.45)
Space group	$P2_12_12_1$	$P2_12_12_1$
Unit cell (Å, º)	43.33 60.52 68.27	43.37 58.97 68.24
Molecules (a.u.)	1	2
Unique reflections	26393 (1257)	62616 (4578)
Completeness (%)	98.8 (97.9)	100 (100)
R_{merge}[b]	0.072 (1.95)	0.112 (2.08)
R_{meas}[c]	0.083 (2.25)	0.116 (2.12)
CC(1/2)	0.998 (0.203)	0.999 (0.571)
Multiplicity	4.3 (4.2)	3.5 (3.6)
I/sig(I)	11.3 (1.1)	12.9 (10.6)
B_{Wilson} (Å^2)	20.4	15.7
R_{work}[e]/R_{free}[f] (%)	21.5/24.7	20/21.9
r.m.s.d. bonds (Å)	0.004	0.006
r.m.s.d. angles (°)	0.731	0.872

[a] Values in parentheses are for the highest resolution shell.

[b] $$R_{merge} = \frac{\sum_{hkl}\sum_{i=1}^{N}\left|I_{i(hkl)} - \bar{I}_{(hkl)}\right|}{\sum_{hkl}\sum_{i=1}^{N} I_{i(hkl)}}$$

[c] $$R_{meas} = \frac{\sum_{hkl}\sqrt{N/(N-1)}\sum_{i=1}^{N}\left|I_{i(hkl)} - \bar{I}_{(hkl)}\right|}{\sum_{hkl}\sum_{i=1}^{N} I_{i(hkl)}}$$

where $\bar{I}_{(hkl)}$ is the mean intensity of multiple $I_{i(hkl)}$ observations of the symmetry-related reflections, N is the redundancy

[e] $$R_{work} = \frac{\sum \left\| F_{obs} \right| - \left| F_{calc} \right\|}{\sum \left| F_{obs} \right|}$$

[f] R_{free} is the cross-validation R_{factor} computed for the randomly chosen test set of reflections (5 %) which are omitted in the refinement process.

Supplementary Table 2. Peptides

	**Sequence**	**Experiment**	**Source**
CTD	Atto488-PSYSPTSPSYSPTSPS	Fluorescence anisotropy	Eurogentec
1xY1P	Atto488-PS**pY**SPTSPSYSPTSPS	Fluorescence anisotropy	Eurogentec
2xY1P	Atto488-PS**pY**SPTSPS**pY**SPTSPS	Fluorescence anisotropy	Eurogentec
1xS2P	Atto488-PSY**pS**PTSPSYSPTSPS	Fluorescence anisotropy	Eurogentec
2xS2P	Atto488-PSY**pS**PTSPSY**pS**PTSPS	Fluorescence anisotropy	Eurogentec
1xT4P	Atto488-PSYSP**pT**SPSYSPTSPS	Fluorescence anisotropy	Eurogentec
2xT4P	Atto488-PSYSP**pT**SPSYSP**pT**SPS	Fluorescence anisotropy	Eurogentec
1xS5P	Atto488-PSYSPT**pS**PSYSPTSPS	Fluorescence anisotropy	Eurogentec
2xS5P	Atto488-PSYSPT**pS**PSYSPT**pS**PS	Fluorescence anisotropy	Eurogentec
1xS7P	Atto488-PSYSPTSP**pS**YSPTSPS	Fluorescence anisotropy	Eurogentec
2xS7P	Atto488-PSYSPTSP**pS**YSPTSP**pS**	Fluorescence anisotropy	Eurogentec
NCoR	FAM-REPAPLLSAQYETLSDSDD	Fluorescence anisotropy	EMC microcollections
NCoR pS2436	FAM-REPAPLLSAQYETL**pS**DSDD	Fluorescence anisotropy	EMC microcollections
FMR1	FAM-SNASETESDHRDELSDWS	Fluorescence anisotropy	Genosphere
FMR1 pS511	FAM-SNASETESDHRDEL**pS**DWS	Fluorescence anisotropy	Genosphere
WTAP	FAM-MTNEEPLPKKVRLSETDFK	Fluorescence anisotropy	Genosphere
WTAP pS14	FAM-MTNEEPLPKKVRL**pS**ETDFK	Fluorescence anisotropy	Genosphere
ZC3H13	LTPPLRRSASPYPSHSLSSP	Fluorescence anisotropy	Genosphere
ZC3H13 pS372	LTPPLRRSA**pS**PYPSHSLSSP	Fluorescence anisotropy	Genosphere
ZC3H13 pS381	LTPPLRRSASPYPSHSLS**pS**P	Fluorescence anisotropy	Genosphere
ZC3H13 pS372 pS381	LTPPLRRSA**pS**PYPSHSLS**pS**P	Fluorescence anisotropy	Genosphere
1xS5P	PSYSPT**pS**PSYSPTSPS	X-ray crystallography	AnaSpec

Supplementary Table 3. Antibodies

	Source	Identifier	
Rabbit Anti-GFP	Abcam	RRID:AB_303395; ab290	ChIP: 6 µL/10^8 cells; 1:1000 for IF 1:1000 for WB
Mouse anti-FLAG M2-peroxidase	Sigma	RRID:AB_439702; A8592	1:10000 for WB; 1:500 for IF
Mouse anti-Pol II clone F-12	Santa Cruz	RRID:AB_630203; sc-55492	1:1000 for WB
Rabbit anti-SPT6	Novus Biologicals	RRID:AB_2196402; NB100-2582	1:1000 for WB
Mouse anti-DSIF	Becton Dickinson	RRID:AB_398420; 611107	1:1000 for WB
Rabbit anti-PAF1	Abcam	RRID:AB_2159769; ab20662	1:1000 for WB
Rabbit anti-Leo1	Bethyl	RRID: AB_309451; A300-174A	1:1000 for WB
Rabbit anti-CK2α	Cell Signaling	RRID:AB_2236816; 2656	1:1000 for WB
Rat anti-ZNF768 (5c8)	Kindly provided by Dirk Eick		1:10 for WB
Rabbit anti-MSH2	Cell Signaling	RRID:AB_2235387; #2017	1:1000 for WB
Mouse anti-HTATSF1	Santa Cruz	RRID:AB_2894929; sc-514351	1:1000 for WB
Rabbit anti-RBM15	Bethyl	RRID:AB_2253435; A300-821A	1:1000 for WB; 1:200 for IF
Rabbit anti-PCNA	Abcam	RRID:AB_444313; Ab18197	1:1000 for WB
Rabbit anti-WTAP	Cell Signaling	RRID:AB_2799512; 56501	1:1000 for WB
Rabbit anti-CHK2	Cell Signaling	RRID:AB_2080793; 2662	1:1000 for WB
Rabbit anti-IWS1	Cell Signaling	RRID:AB_10694503; 5681	1:1000 for WB
Mouse anti-FMR1	Merck Millipore	RRID:AB_2909408; MABN2453	1:1000 for WB
Mouse anti-α-tubulin	Sigma	RRID:AB_477582: T6074	1:5000 for WB
Rabbit anti-DIDO1	Atlas antibodies	RRID: AB_2680944 HPA049904	1:500 for WB
Goat anti-mouse Alexa Fluor 568	Invitrogen	A11004	1:500 for IF
Goat anti-rabbit Alexa Fluor 568	Invitrogen	A11011	1:500 for IF

Supplementary Table 4. Oligonucleotides

	**Sequence**	**Experiment**
CMV10_down	5‘-TCTAGAGGATCCCGGGTGGCATC-3‘	Gibson assembly with CMV10 N3XFLAG
CMV10_up	5‘-CGCAAGCTTGTCATCGTCATCCTTG-3‘	Gibson assembly with CMV10 N3XFLAG
DIDO_down	5‘-CAAGGATGACGATGACAAGCTTGCGATGGACGACAAAGGCGACCCGAGCAATG-3‘	Gibson assembly of DIDO/DIDO dSPOC with CMV10 N3XFLAG
DIDO_up	5‘-GATGCCACCCGGGATCCTCTAGACTAGGCCTGCGAGGCGGTGCC-3‘	Gibson assembly of DIDO/DIDO dSPOC with CMV10 N3XFLAG
DIDO_dSPOC_down	5‘-ACGTTCCCCTCCAGAGGGAGACACGGGAGAGTTAGACAAGATGGACGAAAAGCGG-3‘	Gibson assembly of DIDO dSPOC with CMV10 N3XFLAG
DIDO_dSPOC_up	5‘-CGTGTCTCCCTCTGGAGGGGAACG-3‘	Gibson assembly of DIDO dSPOC with CMV10 N3XFLAG
SHARP_down	5‘-CAAGGATGACGATGACAAGCTTGCGATGGTCCGGGAAACCAGGCATCTCTGGG-3‘	Gibson assembly of SHARP/SHARP dSPOC with CMV10 N3XFLAG
SHARP_up	5‘-GGGATGCCACCCGGGATCCTCTAGATCACACGGAGGCAATGACAATCATGAGGTG-3‘	Gibson assembly of SHARP with CMV10 N3XFLAG
SHARP_dSPOC_up	5‘-GGGATGCCACCCGGGATCCTCTAGATCAGGGTCTCTGGGAAGTCAGGTGTGGAGAG-3‘	Gibson assembly of SHARP dSPOC with CMV10 N3XFLAG
RBM15_NotI_down	5‘-CGTGCGGCGGCCGCGATGAGGACTGCGGGGCGGGAC -3‘	Cloning RBM15/RBM15 dSPOC into CMV10 N3XFLAG
RBM15_XbaI_up	5‘-GCTCTAGACTATAACAGGGTCAGCGCCAAGTTTTC -3‘	Cloning RBM15 into CMV10 N3XFLAG
RBM15_noSPOC_XbaI_up	5‘-GCTCTAGACTAAGGGGCTGTCCCCCCATCCTG-3‘	Cloning RBM15 dSPOC into CMV10 N3XFLAG
PHF3_NLS_SPOC_NotI_fw	5‘-CGTGCGGCGGCCGCGATGCGAGCCCCTAAGAAAAAGCGGAAGGTGGGCGGCTCTACCTTTCTGGCTCGATTG-3‘	Cloning PHF3 NLS-SPOC into CMV10 N3XFLAG
PHF3_SPOC_XbaI_rv	5‘-GCTCTAGATTAACTGTGCTGTCGCTTCAG-3‘	Cloning PHF3 NLS-SPOC into CMV10 N3XFLAG
DIDO_NLS_SPOC_NotI_fw	5‘-CGTGCGGCGGCCGCGATGCGAGCCCCTAAGAAAAAGCGGAAGGTGGGCGGCACCCTCTTTTTGTCTCGACTC-3‘	Cloning DIDO NLS-SPOC into CMV10 N3XFLAG
DIDO_SPOC_XbaI_rv	5‘-GCTCTAGATTAACTGTTTGCGGGACGTTTGAT-3‘	Cloning DIDO NLS-SPOC into CMV10 N3XFLAG
SHARP_NLS_SPOC_NotI_fw	5‘-CGTGCGGCGGCCGCGATGCGAGCCCCTAAGAAAAAGCGGAAGGTGGGCGGCGTGGATATGGTTCAACTTCTGAAG-3‘	Cloning SHARP NLS-SPOC into CMV10 N3XFLAG
SHARP_SPOC_XbaI_rv	5‘-GCTCTAGATCACACGGAGGCAATGACAA-3‘	Cloning SHARP NLS-SPOC into CMV10 N3XFLAG

RBM15_NLS_SPOC_NotI_fw	5‘-CGTGCGGCGGCCGCGATGCGAGCCCCTAAGAAAAAGCGGAAGGTGGGCGGCGCCCCTGTGGCATCAGC-3‘	Cloning RBM15 NLS-SPOC into CMV10 N3XFLAG
RBM15_SPOC_XbaI_rv	5‘-GCTCTAGATTATATCTGAAAACCAAACCCACGG-3‘	Cloning RBM15 NLS-SPOC into CMV10 N3XFLAG
SPOC_DIDO_NcoI_down	5‘-CGTGCGCCATGGGCACCCTCTTTTGTCTCGACTCAGC-3‘	Cloning DIDO SPOC into pET M11
SPOC_DIDO_XhoI_up	5‘-GCCTCGAGTCAACTGTTTGCGGGACGTTTGA-3‘	Cloning DIDO SPOC into pET M11
SHARP_SPOC_NcoI_down	5‘-CGTGCGCCATGGGCGTGGATATGGTTCAACTTCTGAAGAAG-3‘	Cloning SHARP SPOC into pET M11
SHARP_SPOC_XhoI_up	5‘-GCCTCGAGTCACACGGAGGCAATGACAATC-3‘	Cloning SHARP SPOC into pET M11
RBM15_SPOC_NcoI_down	5‘-CGTGCGCCATGGGCGCCCCTGTGGCATCAGCCTC-3‘	Cloning RBM15 SPOC into pET M11
RBM15_SPOC_XhoI_up	5‘-GCCTCGAGTCATATCTGAAAACCAAACCCACGGACAATGATCATGACC-3‘	Cloning RBM15 SPOC into pET M11
SPOCD1_SPOC_NcoI_down	5‘-CGTGCGCCATGGGCACAAAGGCCCTGCCCTGC-3‘	Cloning SPOCD1 into pET M11
SPOCD1_SPOC_XhoI_up	5‘-GCCTCGAGTCATGCTGTGTCTGGAAGCCCTTC-3‘	Cloning SPOCD1 into pET M11
DIDO_R1096A_down	5‘-GGGATCGCACCGAAGACAGTTTGGGATTATGTTGGCAAACTCAAGT-3‘	Site directed mutagenesis
DIDO_R1096A_up	5‘-TCTTCGGTGCGATCCCCCCACCAATGTGAATTGTGTCAGGCAAATC-3‘	Site directed mutagenesis
SHARP_R3552A_down	5‘-CAGGCGATGCGGCTGGAGGCAACGCAGCTGGAAGGGGTTGCCCGAA-3‘	Site directed mutagenesis
SHARP_R3552_up	5‘-CCAGCCGCATCGCCTGGGCGATCCTTAGTGGGGGCCCTCCTTCAGA-3‘	Site directed mutagenesis
RBM15_R834A_down	5‘-CAGGCTCTCCGTTTGGACCAGCCCAAGTTGGATGAAGTAACTCGAC-3‘	Site directed mutagenesis
RBM15_R834A_up	5‘-CCAAACGGAGAGCCTGAGTGATCTTGAGCTGGGCCACTTTGCCTCC-3‘	Site directed mutagenesis
SHARP_3left_fw	5‘-CACCTGACGTCTACCACCATGGCACCACCATG-3’	Cloning repair template for SHARP-GFP
SHARP_3left_rev	5‘-GACCGCTCGACGACACGGAGGCAATGACAATCATG-3‘	Cloning repair template for SHARP-GFP
SHARP 3right_fw	5‘-GCCCGGTGCCTGAGCCACTGAGTGGTTATCAC-3‘	Cloning repair templates for SHARP-GFP and SHARP ΔSPOC-GFP

SHARP 3right_rev	5‘-GTTCTTTCCTGCGACAGTTTCATAAATTAATAAGTGTTAGG-3‘	Cloning repair templates for SHARP-GFP and SHARP ΔSPOC-GFP
SHARP SPOCleft_fw	5‘-CACCTGACGTCTAGTCTGTTGGGCATGTGCTTG-3‘	Cloning repair template for SHARP ΔSPOC-GFP
SHARP SPOCleft_rev	5‘-GACCGCTCGACGAGGGTCTCTGGGAAGTCAG-3‘	Cloning repair template for SHARP ΔSPOC-GFP
vector 3SHARP_fw	5‘-TTTATGAAACTGTCGCAGGAAAGAACATGTGAG-3‘	Cloning repair templates for SHARP-GFP and SHARP ΔSPOC-GFP
vector 3SHARP_rev	5‘-GGTGCCATGGTGGTAGACGTCAGGTGGCACTTTTC-3‘	Cloning repair template for SHARP-GFP
vector SPOC_SHARP_rev	5‘-ATGCCCAACAGACTAGACGTCAGGTGGCACTTTTC-3‘	Cloning repair template for SHARP ΔSPOC-GFP
SHARP 3GFP_fw	5‘-CATTGCCTCCGTGTCGTCGAGCGGTCCCTCG-3‘	Cloning repair template for SHARP-GFP
SHARP 3GFP_rev	5‘-ACCACTCAGTGGCTCAGGCACCGGGCTTGCG-3‘	Cloning repair templates for SHARP-GFP and SHARP ΔSPOC-GFP
SHARP SPOC_GFP_fw	5‘-TTCCCAGAGACCCTCGTCGAGCGGTCCCTCG-3‘	Cloning repair template for SHARP ΔSPOC-GFP
DIDO SPOCleft_fwd	5‘-TAGAAAGTGCTTCTCATCCAAATGT-3‘	Cloning repair template for DIDO ΔSPOC
DIDO SPOCleft_rev	5‘-CGAGCTGTACAAGTAAATAACTTCGTATAATGTATGCTATACGAAGTTATAGTAGAAAGCCTTTTTTTTTTTGAGACAG-3‘	Cloning repair template for DIDO ΔSPOC
DIDO SPOCright_fwd	5‘-TCTTTTATTTTATCGGATAACTTCGTATAGCATACATTATACGAAGTTATTTTGAAATTCTCATTGCACAGAGAGAC-3‘	Cloning repair template for DIDO ΔSPOC
DIDO SPOCright_rev	5‘-TTCTTCTTGCTCCTCCAGCT-3‘	Cloning repair template for DIDO ΔSPOC
vector SPOC_DIDO_fwd	5‘-GAGACAGCTGGAGGAGCAAGAAGAAAGACATTAGGTGGAGTTCAG-3‘	Cloning repair template for DIDO ΔSPOC
vector SPOC_DIDO_rev	5‘-ACATTTGGATGAGAAGCACTTTCTATCGTACGATGGGTTTTGTTTC-3‘	Cloning repair template for DIDO ΔSPOC
RBM15 SPOCleft_fwd	5‘-CCTATCAAAATTGGTTATGGTAAAGCTACA-3‘	Cloning repair template for RBM15 ΔSPOC
RBM15 SPOCleft_rev	5‘-CGAGCTGTACAAGTAAATAACTTCGTATAATGTATGCTATACGAAGTTATCTATGTCCCCCCATCCTGTT-3‘	Cloning repair template for RBM15 ΔSPOC
RBM15 SPOCright_fwd	5‘-TCTTTTATTTTATCGGATAACTTCGTATAGCATACATTATACGAAGTTATTGGTTATAGTGGTGTCCCTA-3‘	Cloning repair template for RBM15 ΔSPOC
RBM15 SPOCright_rev	5‘-ACCGGAGCCAATTCCATAACTTCGTATAGCATACATTATACGAAGTTATTGGTTATAGTGGTGTCCCTA-3‘	Cloning repair template for RBM15 ΔSPOC

vector SPOC_RBM15_fwd	5‘-CTCACACAGCTTAAGAGTAGCTGTCAGACATTAGGTGGAGTTCAG-3‘	Cloning repair template for RBM15 ΔSPOC
vector SPOC_RBM15_rev	5‘-CTTTACCATAACCAATTTTGATAGGTCGTACGATGGGTTTTGTTTC-3‘	Cloning repair template for RBM15 ΔSPOC
resistance cassette_fwd	5‘-CGAAGTTATTTACTTGTACAGCTCG-3‘	Cloning repair template for DIDO and RBM15 ΔSPOC
resistance cassette_rev	5‘-CGAAGTTATCCGATAAAATAAAAGA-3‘	Cloning repair template for DIDO and RBM15 ΔSPOC
BEX5_fw1	5‘-GTGCAGCCGATTTCAAGGCT-3‘	ChIP-qPCR
BEX5_rev1	5‘-TTCTCCTGCACTCAACTCGG-3‘	ChIP-qPCR
BEX5_fw2	5‘-GTGCCCAATAGGCTTGTCG-3‘	ChIP-qPCR
BEX5_rev2	5‘-GGTCCCCTATAAGAATGCGC-3‘	ChIP-qPCR
HOXA5_fw1	5‘-TACGGCTACGGCTACAATGG-3‘	ChIP-qPCR
HOXA5_rev1	5‘-ATCGGGCTGAGGAGAGTGCG-3‘	ChIP-qPCR
HOXA5_fw2	5‘-TCCCTGCTCATGACCCAAGC-3‘	ChIP-qPCR
HOXA5_rev2	5‘-CAGCTCCAGGGTCTGGTAGC-3‘	ChIP-qPCR
XIST_fw1	5‘-CCCATCGGGGTGACGG-3‘	ChIP-qPCR
XIST_rev2	5‘-AAAAGCACCGATGGGCGATG-3‘	ChIP-qPCR
XIST_fw2	5‘-TGTCACGTGGACATCATGGC-3‘	ChIP-qPCR
XIST_rev2	5‘-GGCTGTGATCAATTCCACCC-3‘	ChIP-qPCR
XIST_fw3	5‘-ACACACAGCTCAACCTATCTGA-3‘	ChIP-qPCR
XIST_rev3	5‘-CAGGAACCGGGACAAACA-3‘	ChIP-qPCR

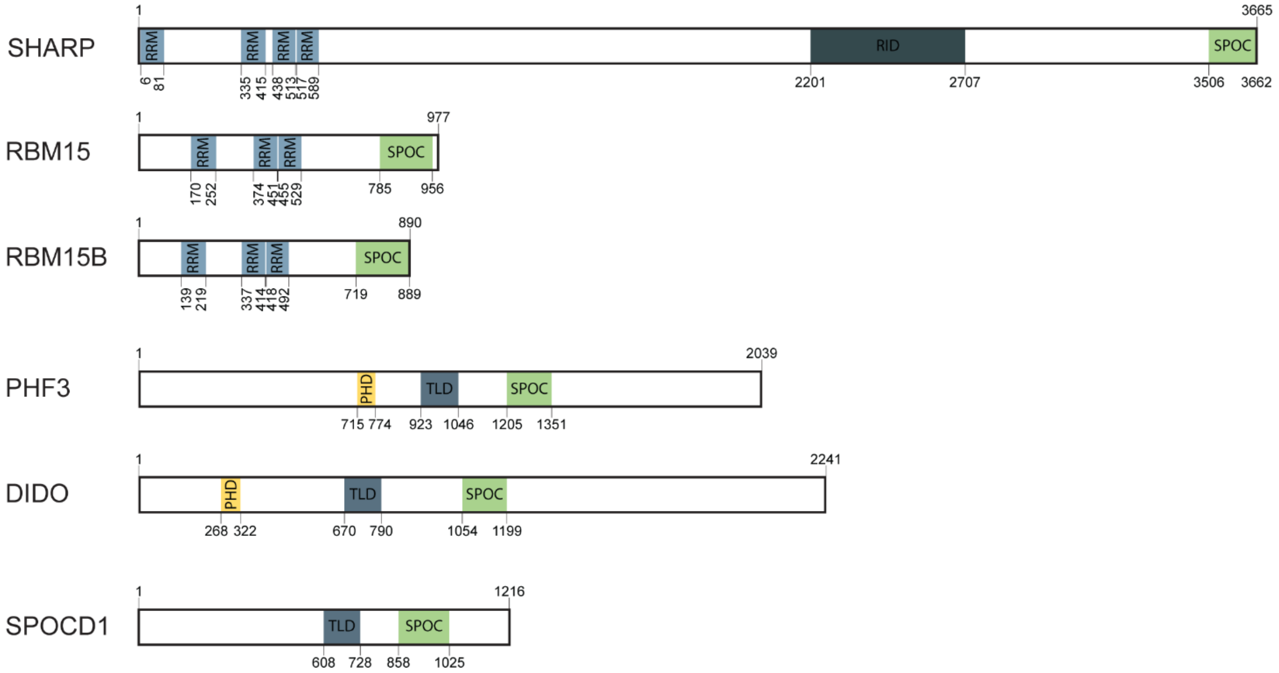

Supplementary Fig. 1: Domain organization of human SPOC proteins. SPOC – Spen orthologue and paralogue C-terminal domain, RRM – RNA recognition motif, RID – receptor interaction domain, PHD – plant homeodomain, TLD – transcription factor IIS-like domain.

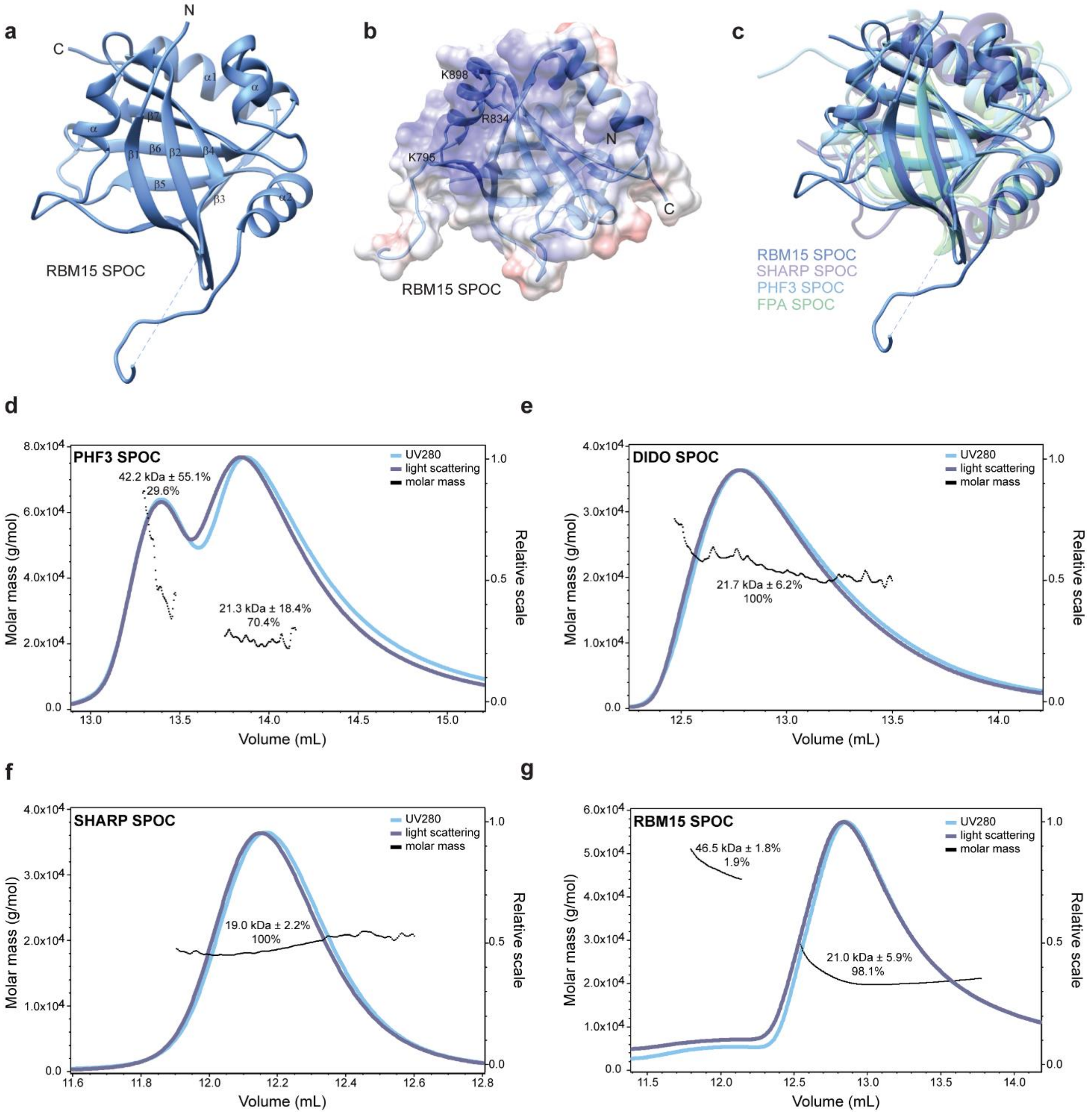

Supplementary Fig. 2: Crystal structure of the RBM15 SPOC domain and SEC-MALS analysis of the oligomeric state of SPOC domains. a, Ribbon model of the crystal structure of RBM15 SPOC. **b** Overlay of RBM15 SPOC ribbon model and electrostatic surface potential. Conserved lysine and arginine residues of the basic patch are indicated. Electrostatic surface potential was calculated using the Coulombic Surface Coloring tool in UCSF Chimera and is depicted ranging from -10 (red) to +10 (blue) kcal/(mol*e). **c** Overlay of SPOC structures from RBM15 (7Z27), SHARP (2RT5), PHF3 (6Q2V) and FPA (5KXF) showed an average RMSD of 1.075 Å over 99 pruned Cα atoms between RBM15 and SHARP SPOC (6.645 Å over all 159 pairs), an average RMSD of 1.075 Å over 72 pruned Cα atoms between RBM15 and PHF3 SPOC (6.387 Å over all 145 pairs) and an average RMSD of 0.913 Å over 53 pruned Cα atoms between RBM15 and FPA SPOC (5.531 Å over all 118 pairs). The alignment was generated using the Matchmaker tool in UCSF Chimera. **d-g** Size exclusion chromatography (SEC)-multiangle light scattering (MALS) profiles of SPOC domains in 25 mM Tris-HCl pH 7.4, 25 mM NaCl, 1 mM DTT, yielding molecular weights of **d** 21.3 kDa ± 18.4% (monomer, 70.4%) and 42.2 kDa ± 55.1% (dimer/trimer, 29.6%) for PHF3 SPOC (expected monomeric Mw 17.8 kDa), **e** 21.7 kDa ± 6.2% (monomer, 100%) for DIDO SPOC (expected monomeric Mw 17.8 kDa), **f** 19.0 kDa ± 2.2% (monomer, 100%) for SHARP SPOC (expected monomeric Mw 18.6 kDa) and **g** 21.0 kDa ± 5.9% (monomer, 98.1%) and 46.5 kDa ± 1.8% (dimer, 1.9%) for RBM15 SPOC.

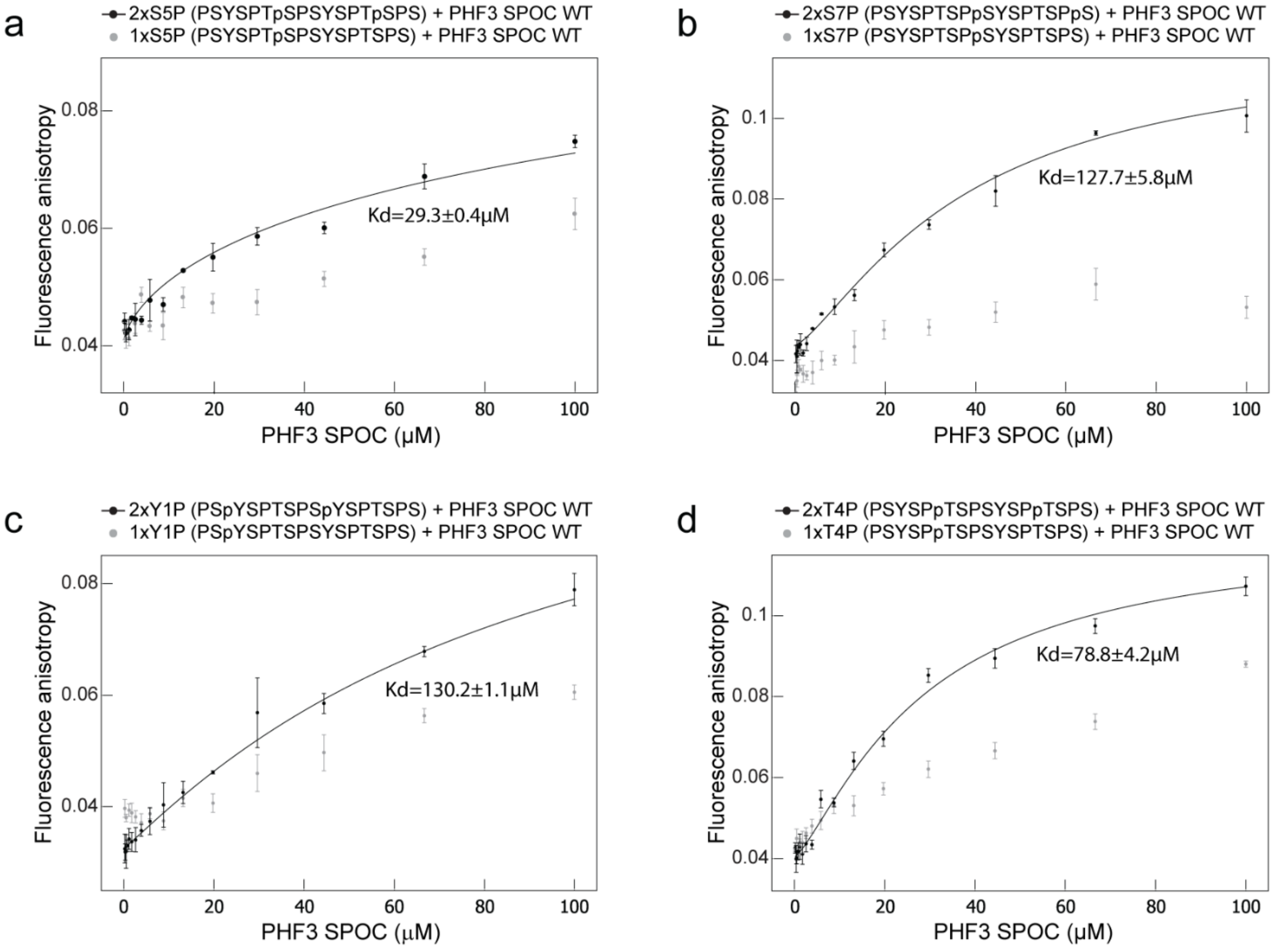

Supplementary Fig. 3: PHF3 SPOC-CTD binding assays. Fluorescence anisotropy measurements of PHF3 SPOC with CTD peptides phosphorylated on **a** serine-5, **b** serine-7, **c** tyrosine-1 and **d** threonine-4. Fluorescence anisotropy is plotted as a function of protein concentration. Data points and error bars represent the mean ± standard deviation from 3 independent experiments. Source data are provided as a Source Data file.

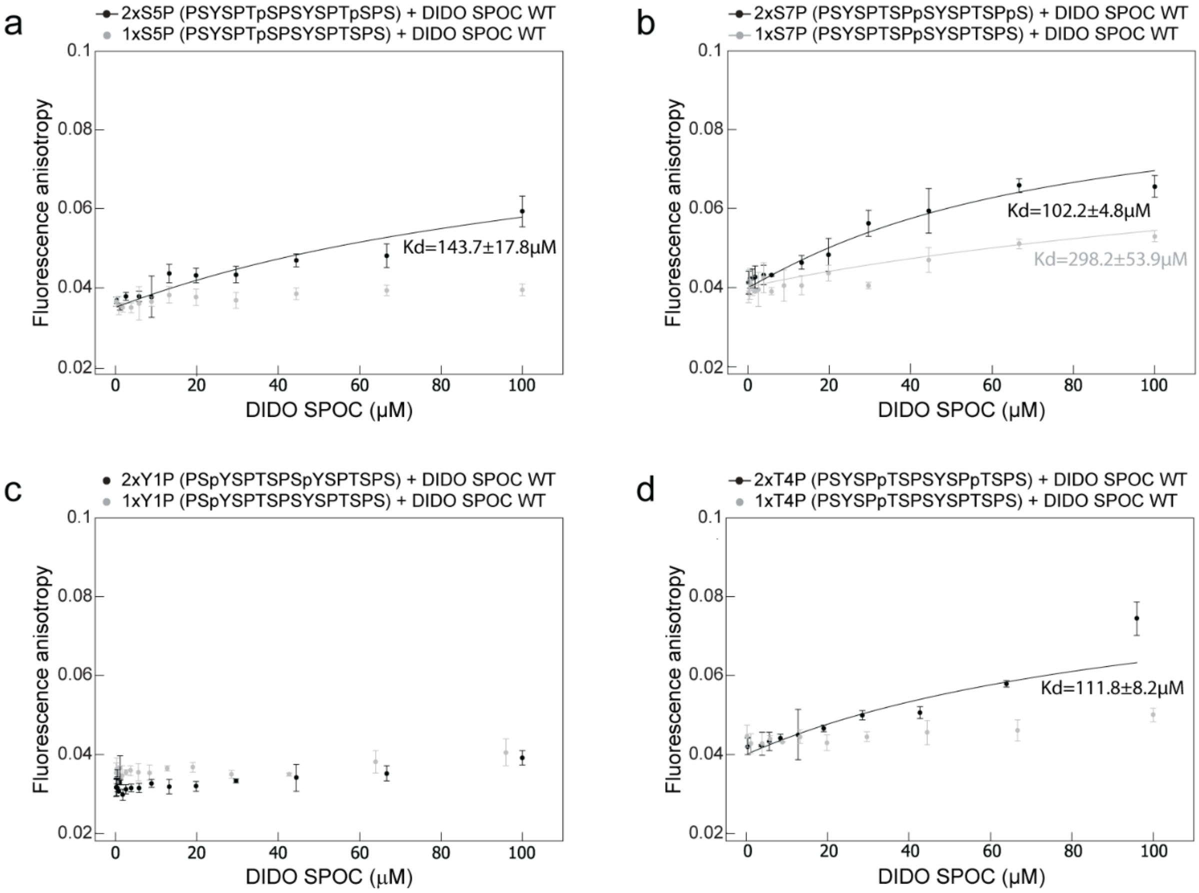

Supplementary Fig. 4: DIDO SPOC-CTD binding assays. Fluorescence anisotropy measurements of DIDO SPOC with CTD peptides phosphorylated on **a** serine-5, **b** serine-7, **c** tyrosine-1 and **d** threonine-4. Fluorescence anisotropy is plotted as a function of protein concentration. Data points and error bars represent the mean ± standard deviation from 3 independent experiments. Source data are provided as a Source Data file.

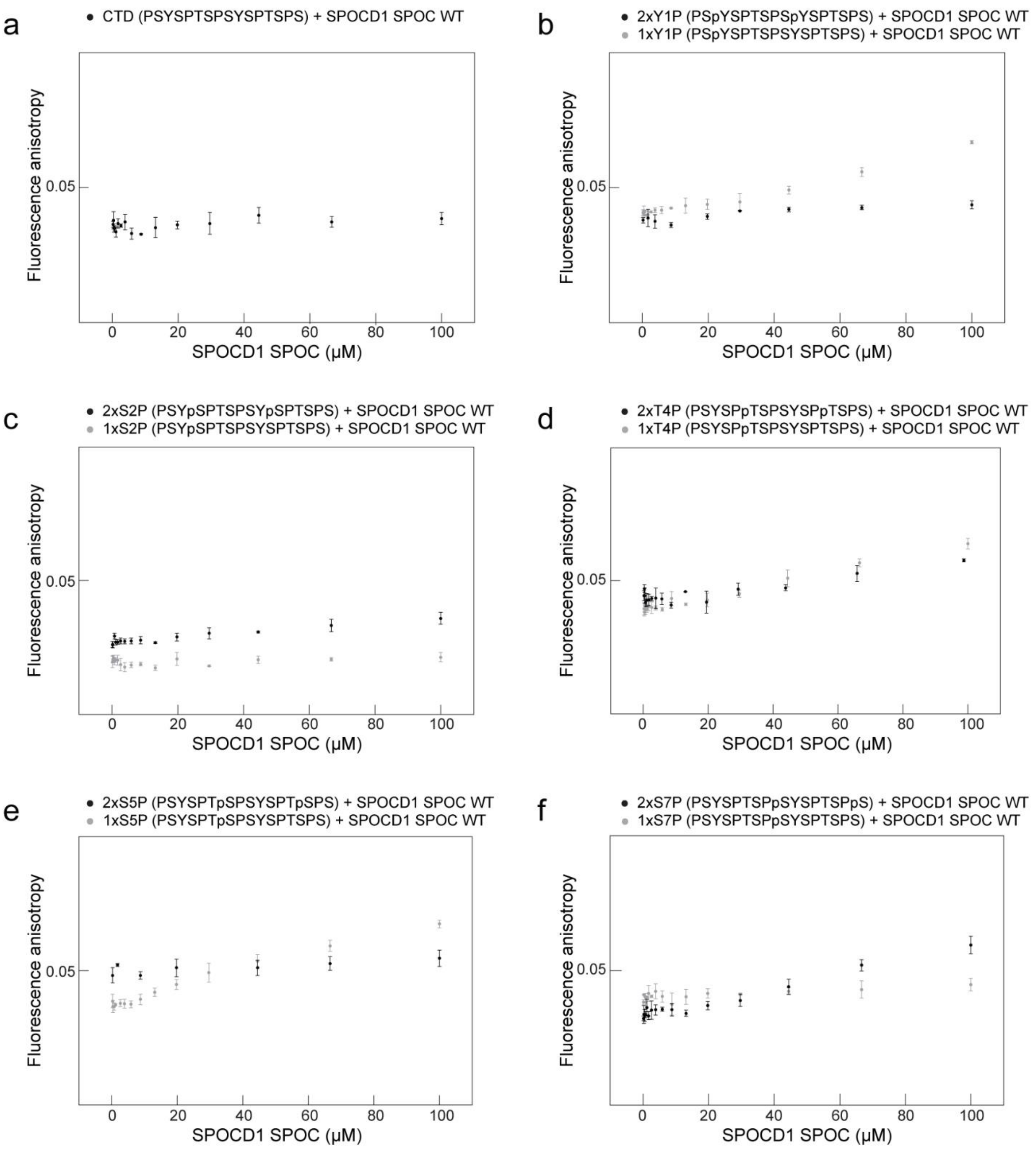

Supplementary Fig. 5: SPOCD1 SPOC-CTD binding assays. Fluorescence anisotropy measurements of SPOCD1 SPOC with **a** unphosphorylated CTD peptide or CTD peptides phosphorylated on **b** tyrosine-1, **c** serine-2, **d** threonine-4, **e** serine-5 and **f** serine-7. Fluorescence anisotropy is plotted as a function of protein concentration. Data points and error bars represent the mean ± standard deviation from 3 independent experiments. Source data are provided as a Source Data file.

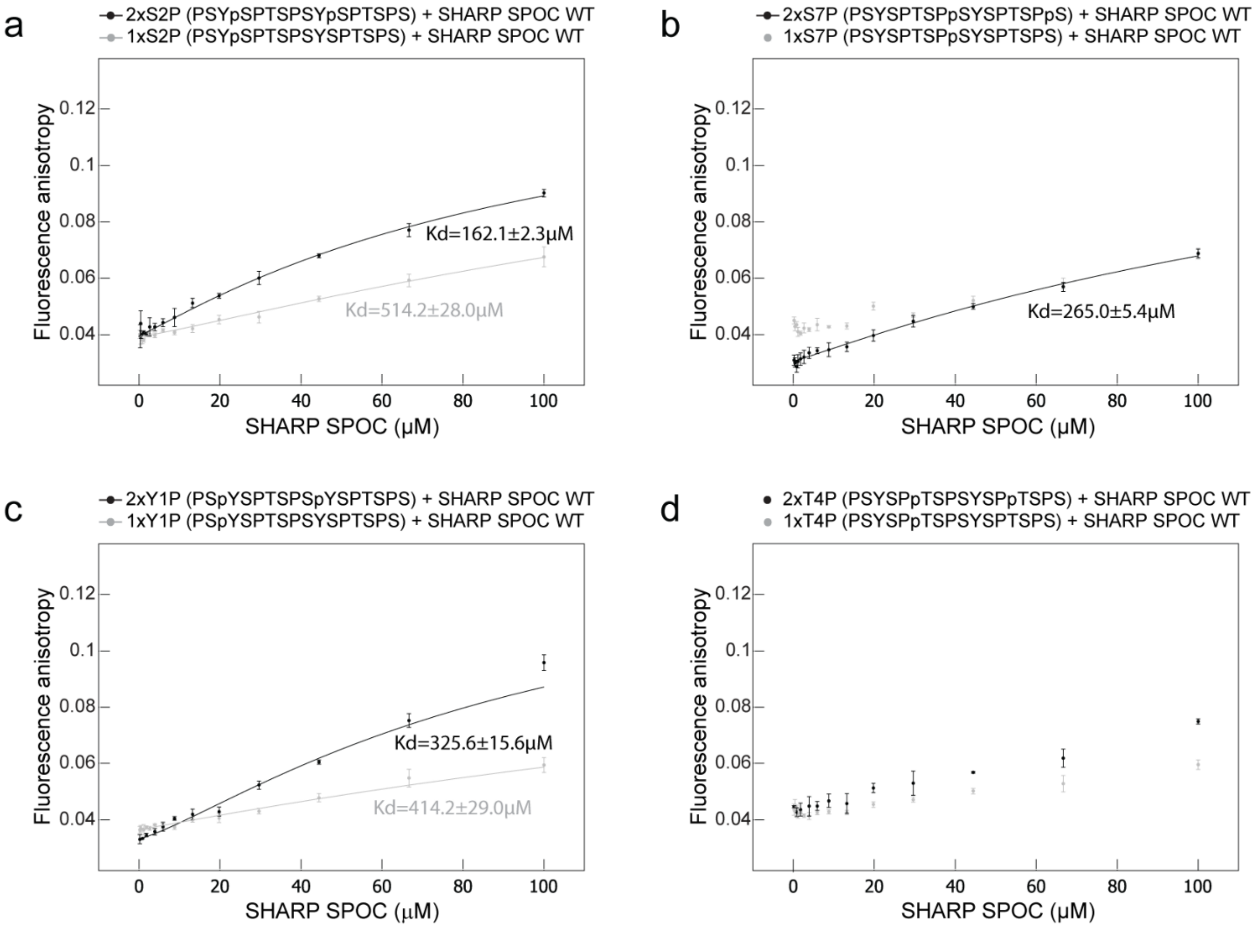

Supplementary Fig. 6: SHARP SPOC-CTD binding assays. Fluorescence anisotropy measurements of SHARP SPOC with CTD peptides phosphorylated on **a** serine-2, **b** serine-7, **c** tyrosine-1 and **d** threonine-4. Fluorescence anisotropy is plotted as a function of protein concentration. Data points and error bars represent the mean ± standard deviation from 3 independent experiments. Source data are provided as a Source Data file.

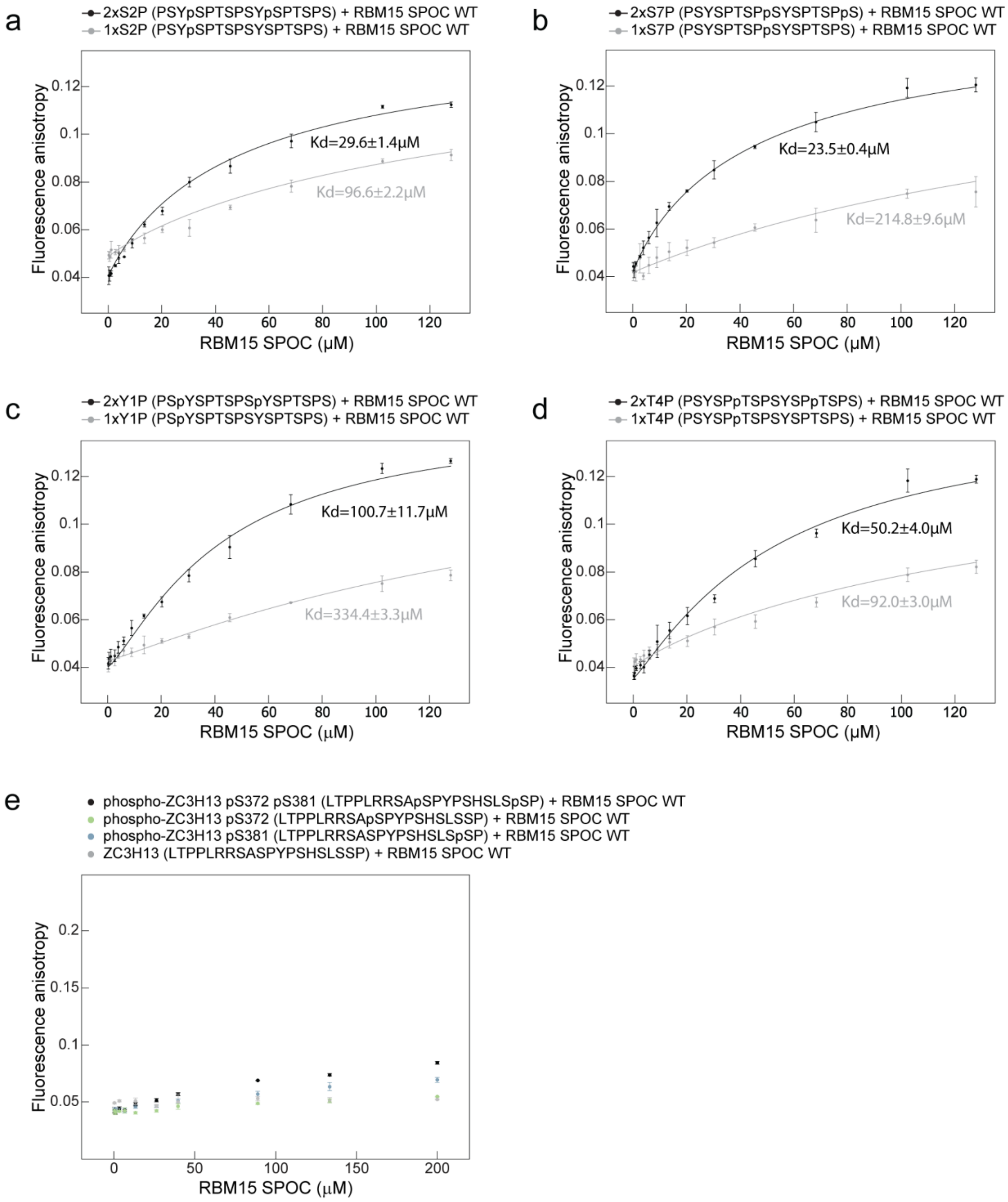

Supplementary Fig. 7: RBM15 SPOC-CTD and RBM15 SPOC-ZC3H13 binding assays. a-d Fluorescence anisotropy measurements of RBM15 SPOC with CTD peptides phosphorylated on **a** serine-2, **b** serine-7, **c** tyrosine-1 and **d** threonine-4. **e** Fluorescence anisotropy measurements of RBM15 SPOC with ZC3H13 peptides. Fluorescence anisotropy in **a-e** is plotted as a function of protein concentration. Data points and error bars represent the mean ± standard deviation from 3 independent experiments. Source data are provided as a Source Data file.

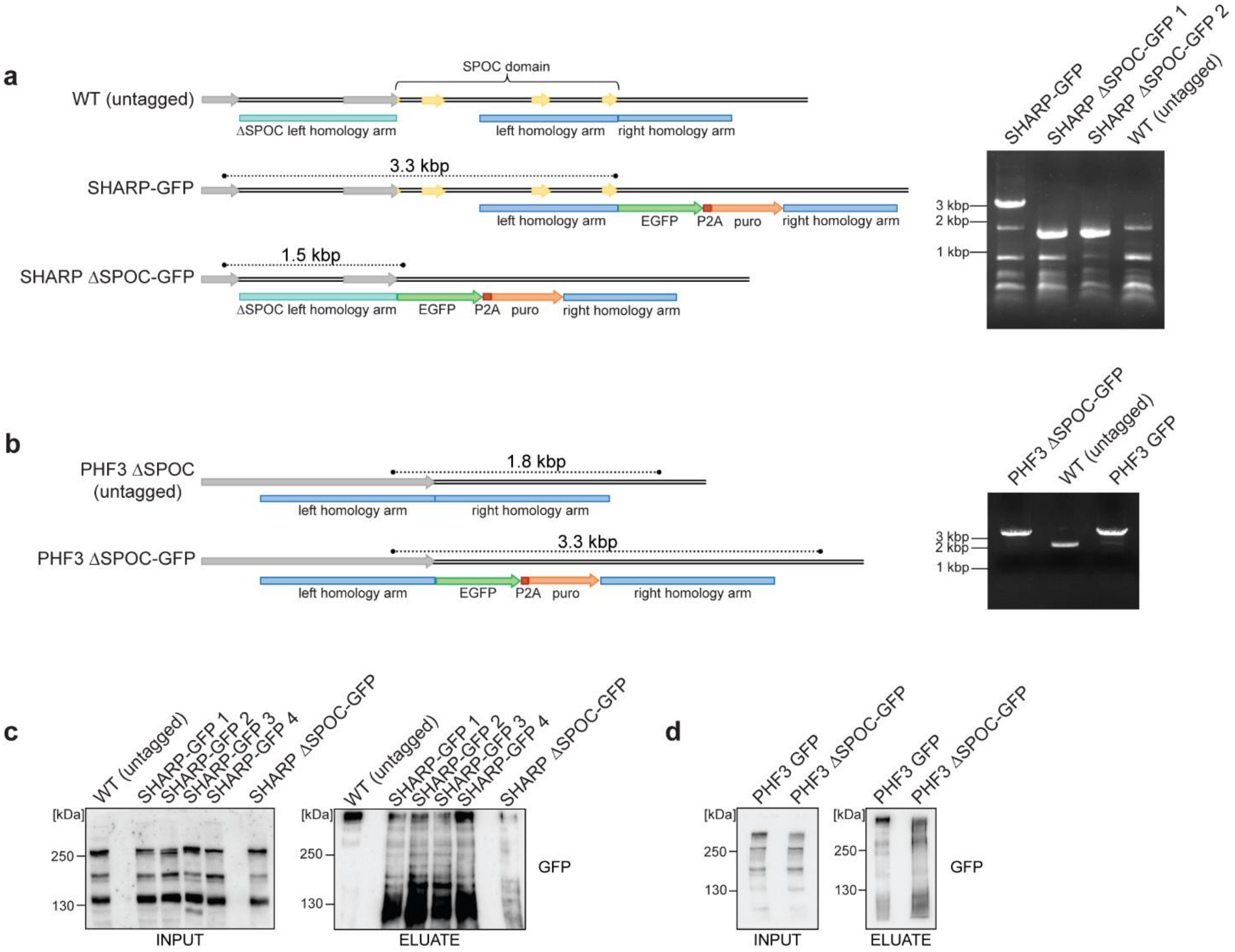

Supplementary Fig. 8: Generation of endogenously tagged SHARP-GFP and PHF3-GFP cell lines. **a** CRISPR/Cas9 strategy and PCR validation of endogenous tagging of SHARP with GFP at the C-terminus (SHARP-GFP) and deletion of the SPOC domain with simultaneous C-terminal GFP-tagging (SHARP ΔSPOC-GFP). The experiment was performed once. Genotyping is shown for two individual SHARP ΔSPOC-GFP clones. **b** CRISPR/Cas9 strategy and PCR validation of endogenous tagging of PHF3 ΔSPOC with GFP at the C-terminus (PHF3 ΔSPOC-GFP). The experiment was performed once. The PHF3 ΔSPOC cell line had been generated and validated before. **c** GFP-IP of endogenous SHARP-GFP and SHARP ΔSPOC-GFP. The experiment was performed once. Four individual clones are shown for SHARP-GFP. **d** GFP-IP of endogenous PHF3-GFP and PHF3 ΔSPOC-GFP. The experiment was performed once. Source data are provided as a Source Data file.

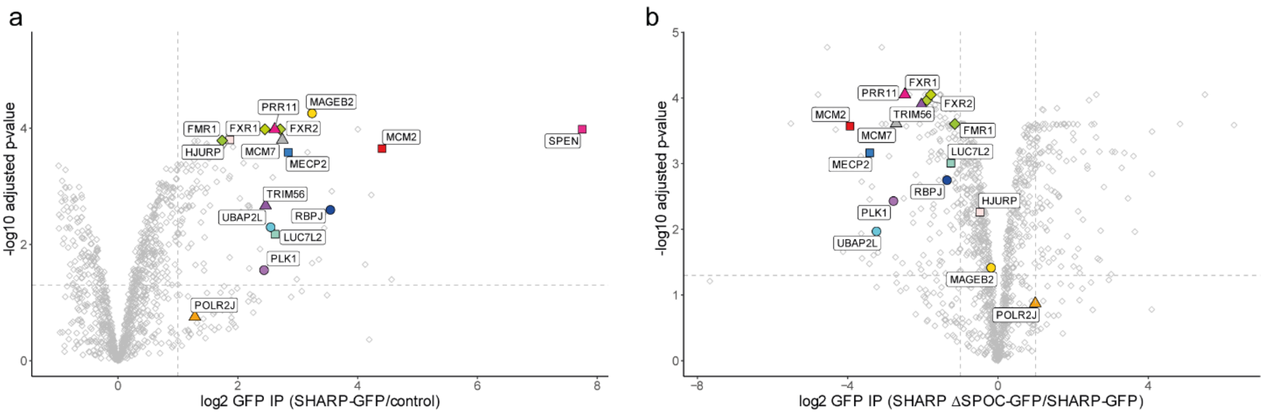

Supplementary Fig. 9: Interactome of endogenous SHARP-GFP. Volcano plots of SHARP-GFP interactors identified by mass spectrometry **a** compared to an untagged control cell line and **b** compared to SHARP ΔSPOC-GFP. The experiments were performed in three replicates. Statistical tests were performed using the LIMMA package[1].

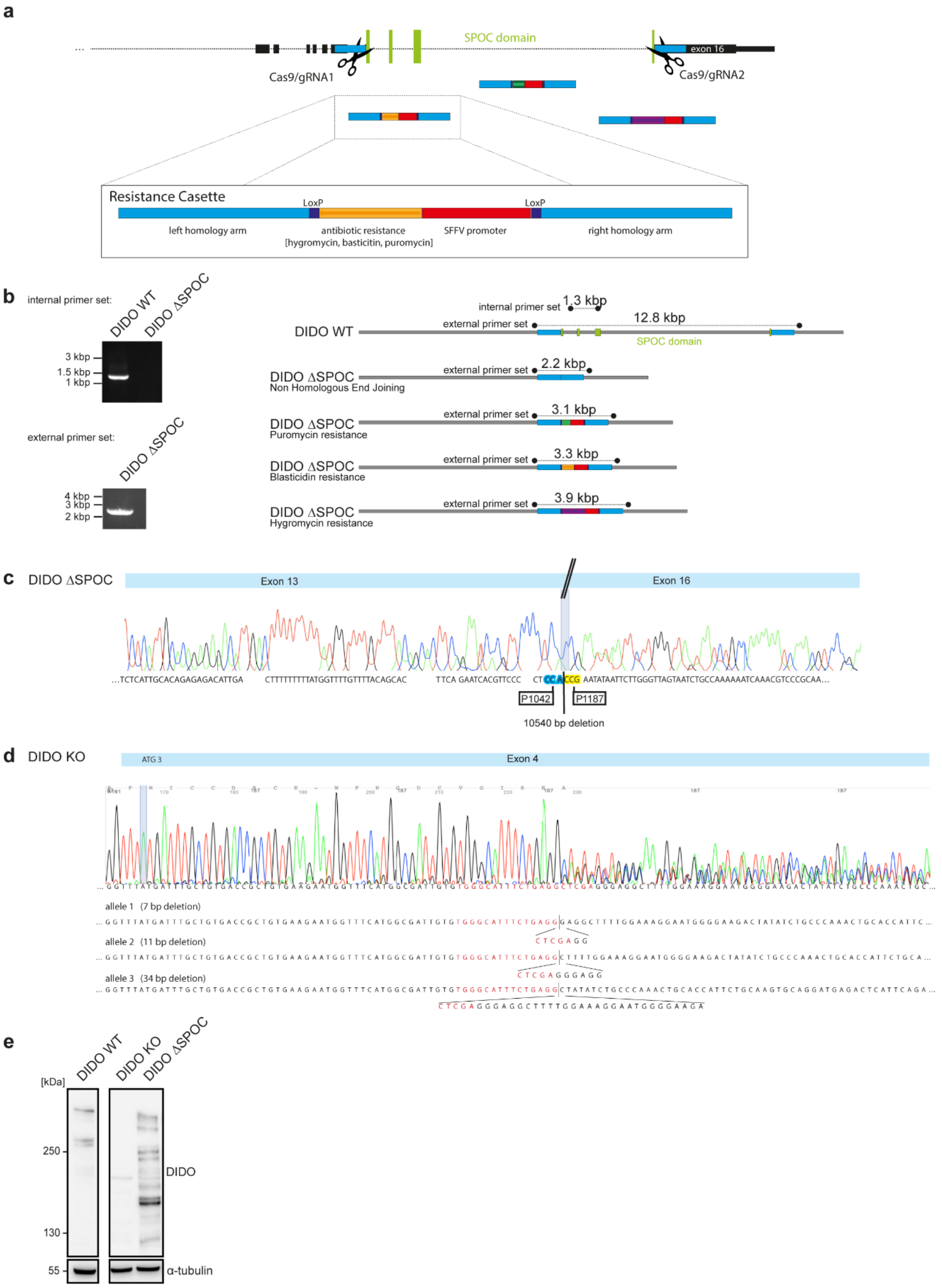

Supplementary Fig. 10: Generation of DIDO ΔSPOC and DIDO KO cell lines. a CRISPR/Cas9 strategy for generation of DIDO ΔSPOC. **b** PCR genotyping strategy and results for DIDO ΔSPOC generation. **c** Validation of DIDO ΔSPOC by Sanger sequencing. **d** Validation of DIDO KO by Sanger sequencing. **e** Western Blot of DIDO WT, KO and ΔSPOC. The experiments in **b** and **e** were performed once. Source data are provided as a Source Data file.

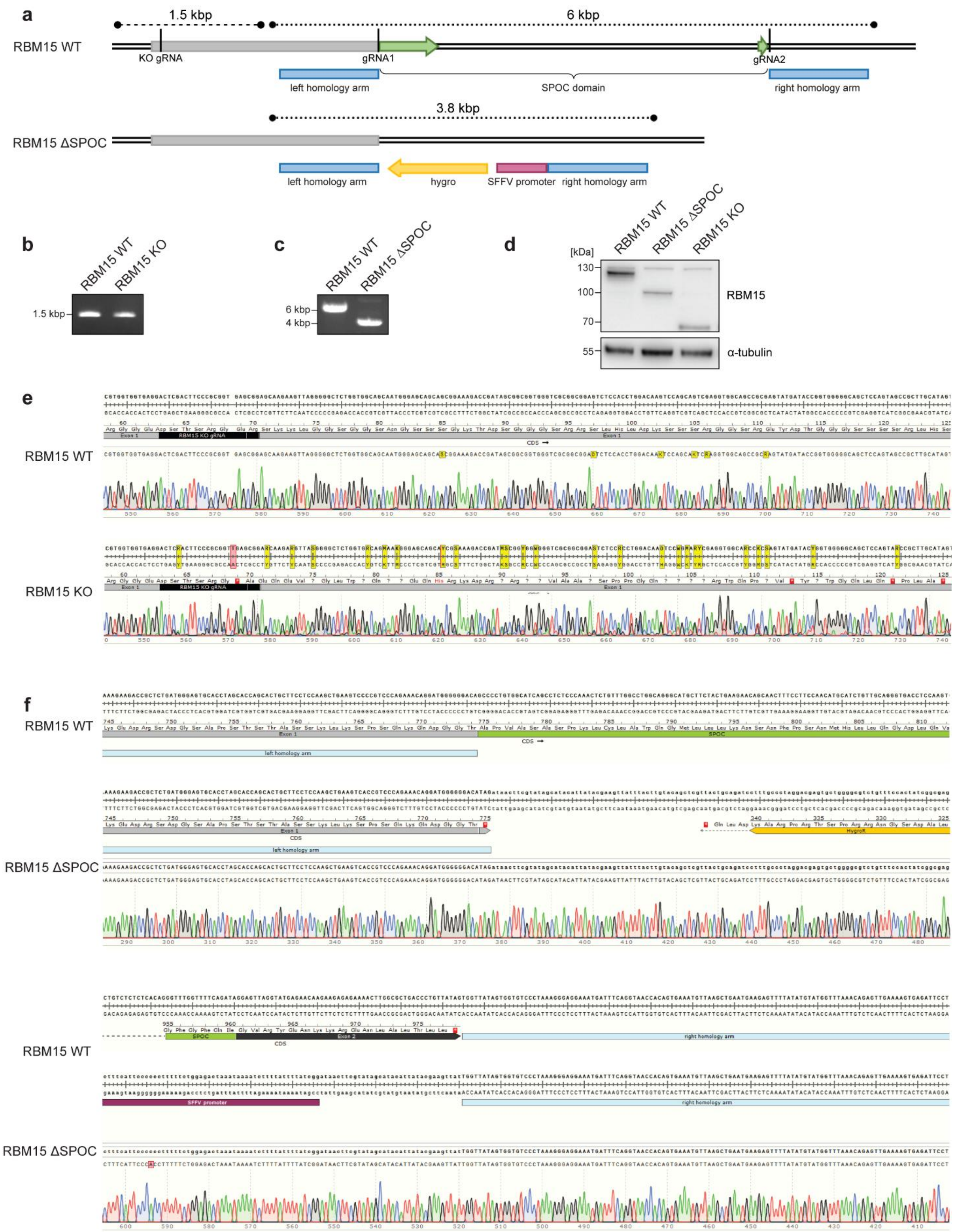

Supplementary Fig. 11: Generation of RBM15 KO and RBM15 ΔSPOC cell lines. a CRISPR/Cas9 strategy for generation and genotyping of RBM15 KO and RBM15 ΔSPOC cell lines. Genotyping PCR products are indicated with dashed (RBM15 KO) or dotted (RBM15 ΔSPOC) lines. **b, c** Genotyping PCR products for **b** RBM15 KO and **c** RBM15 ΔSPOC. **d** Western blot of RBM15 WT, ΔSPOC and KO. **e, f** Validation of **e** RBM15 WT and KO and **f** RBM15 ΔSPOC by Sanger sequencing. The experiments in **b-d** were performed once. Source data are provided as a Source Data file.

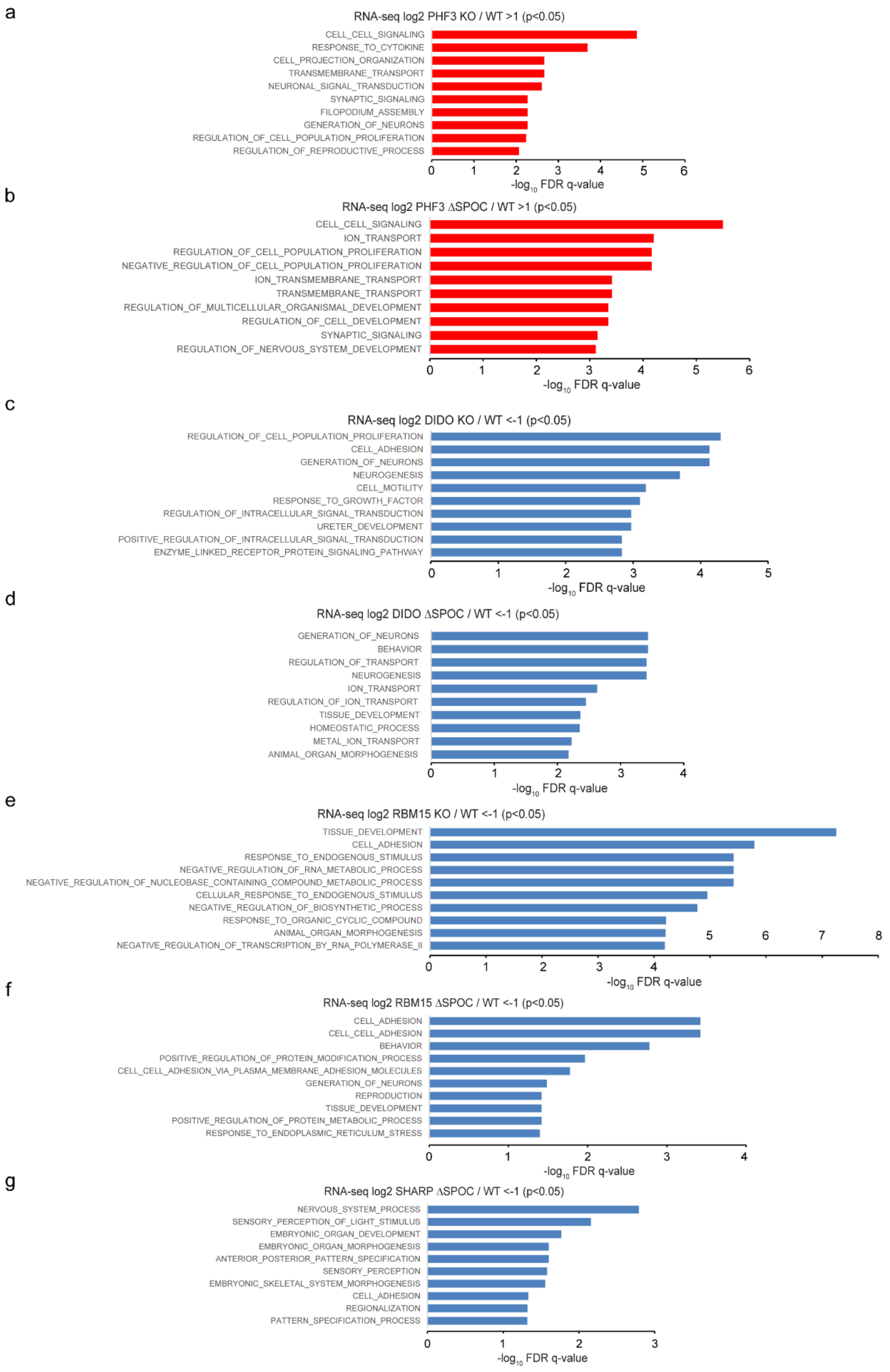

Supplementary Fig. 12: GO analysis of RNA-seq deregulated genes in KO and ΔSPOC HEK293T cells. a Upregulated genes in PHF3 KO, **b** upregulated genes in PHF3 ΔSPOC, **c** downregulated genes in DIDO KO, **d** downregulated genes in DIDO ΔSPOC, **e** downregulated genes in RBM15 KO, **f** downregulated genes in RBM15 ΔSPOC and **g** downregulated genes in SHARP ΔSPOC. GSEA Biological processes tool was used. Top 150 deregulated genes were analysed. FDR q-values were computed by GSEA based on Subramanian et al[2].

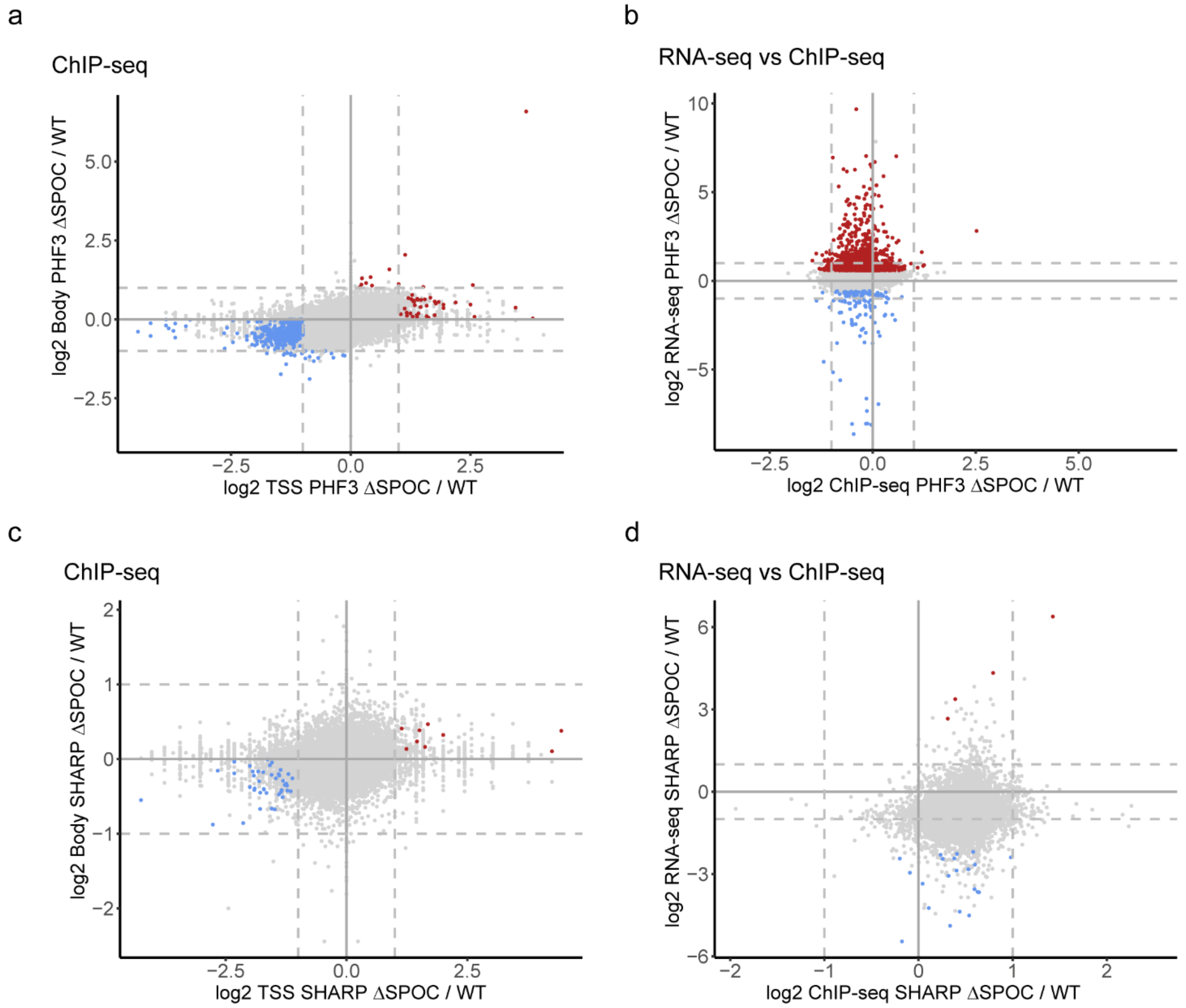

Supplementary Fig. 13: ChIP-seq analysis of the changes in chromatin occupancy upon loss of the SPOC domain in PHF3 and SHARP in HEK293T cells. a,c Relationship between ChIP-seq body fold change and ChIP-seq TSS fold change for **a** PHF3 ΔSPOC-GFP vs PHF3-GFP WT (N=3) and **c** SHARP ΔSPOC-GFP vs SHARP-GFP WT (N=2). Blue and red dots indicate genes with reduced or increased genomic occupancy respectively. **b,d** Relationship between RNA-seq fold change and ChIP-seq body fold change for **b** PHF3 ΔSPOC vs PHF3 WT and **d** SHARP ΔSPOC vs SHARP WT. Blue and red dots indicate genes with reduced or increased RNA-seq gene expression levels respectively.

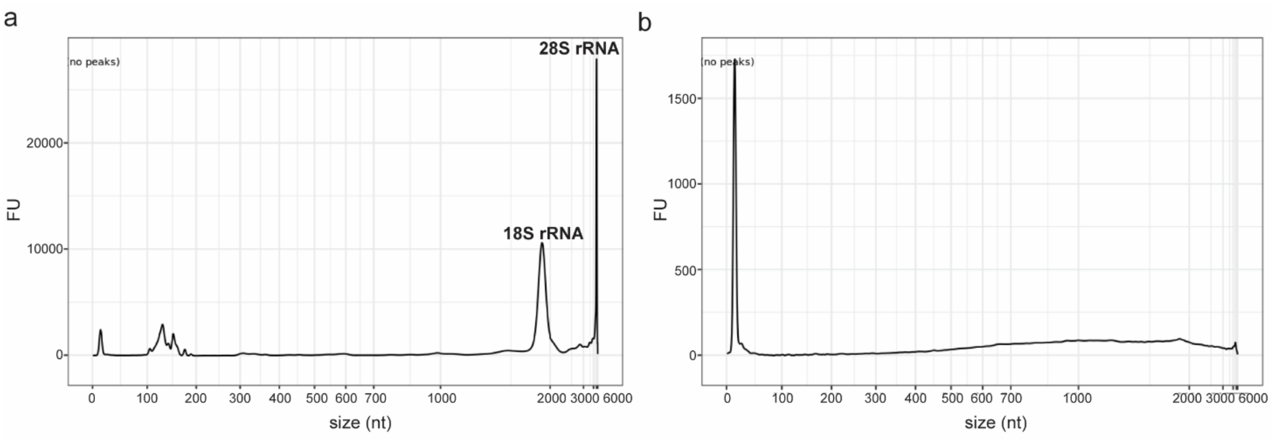

Supplementary Fig. 14: Isolation of mRNA for m^{6}A mass spectrometry analysis. a,b Representative Fragment Analyzer profiles of **a** total RNA and **b** isolated mRNA.

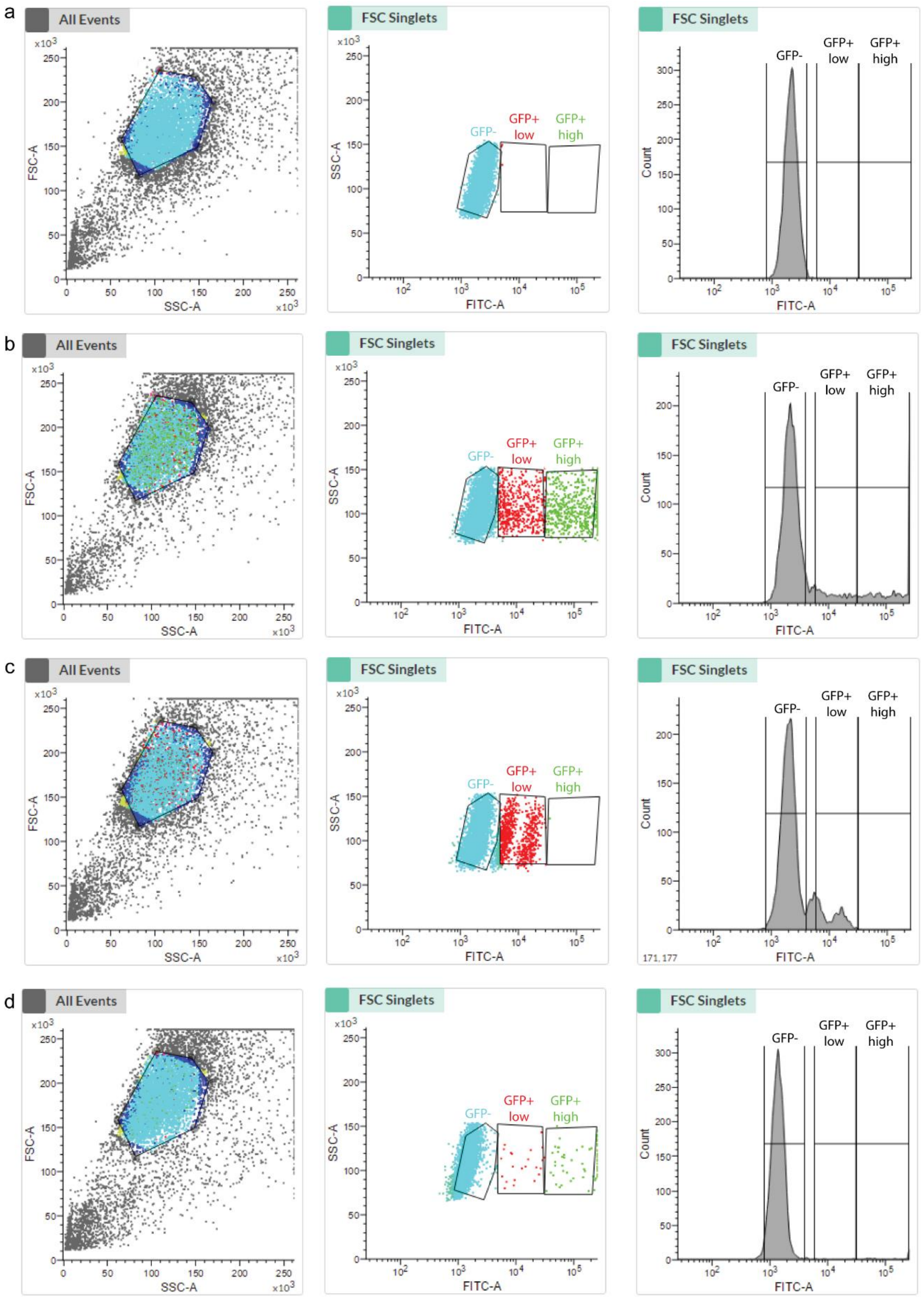

Supplementary Fig. 15: Gating strategy for FACS sorting during cell line generation. Singlet population was defined by forward versus side scatter (FSC vs. SSC) gating. Within the singlet population, three populations were gated based on their FITC/GFP fluorescence: GFP- (negative), GFP+ low (endogenous GFP expression) and GFP+ high (exogenous GFP expression). Histograms depict the distribution of cells over the GFP-groups. Exemplary plots are shown for **a** negative control (untransfected HEK293T cells), **b** HEK293T cells transfected with a pX458 Cas9-EGFP plasmid (GFP+ high cells were sorted), **c** HEK293T cells transfected for endogenous GFP-tagging (GFP+ low cells were sorted) and **d** HEK293T cells 1 week after transfection with a pX458 plasmid (GFP- cells were sorted). 2000 events are depicted in each plot**.**

Supplementary References

1 Ritchie, M. E. *et al.* limma powers differential expression analyses for RNA-sequencing and microarray studies. *Nucleic acids research* **43**, e47, doi:10.1093/nar/gkv007 (2015).

2 Subramanian, A. *et al.* Gene set enrichment analysis: a knowledge-based approach for interpreting genome-wide expression profiles. *Proceedings of the National Academy of Sciences of the United States of America* **102**, 15545-15550, doi:10.1073/pnas.0506580102 (2005).
