# Supplementary material for: The SPOC domain is a phosphoserine binding module that bridges transcription machinery with co- and post-transcriptional regulators
Source: Nat Commun. 2023 Jan 11;14:166. doi: 10.1038/s41467-023-35853-1 (PMC9834408; doi:10.1038/s41467-023-35853-1)
Supplement: Supplementary file 3 — Description of Additional Supplementary Files [file 41467_2023_35853_MOESM3_ESM.pdf]

Description of Additional Supplementary Files

File Name: Supplementary Data 1
Description: Mass spectrometry analysis of the interactome of DIDO, PHF3, RBM15 and SHARP SPOC domains in HEK293T cells.

File Name: Supplementary Data 2
Description: Mass spectrometry analysis of the interactome of full length SHARP and SHARP ΔSPOC in HEK293T cells.

File Name: Supplementary Data 3
Description: RNA-seq analysis in DIDO ΔSPOC, DIDO KO, DIDO WT, RBM15 ΔSPOC, RBM15 KO, RBM15 WT, SHARP ΔSPOC, SHARP WT, PHF3 ΔSPOC, PHF3 KO and PHF3 WT HEK293T cells.

File Name: Supplementary Data 4
Description: TT-seq analysis in DIDO ΔSPOC, DIDO KO, DIDO WT, RBM15 ΔSPOC, RBM15 KO, RBM15 WT, SHARP ΔSPOC, SHARP WT, PHF3 ΔSPOC, PHF3 KO and PHF3 WT HEK293T cells.

File Name: Supplementary Data 5
Description: ChIP-seq analysis.

File Name: Supplementary Data 6
Description: Optimized parameters for each compound analysed in the mass spectrometry analysis of hydrolysed nucleosides.
