# Supplementary material for: The SPOC domain is a phosphoserine binding module that bridges transcription machinery with co- and post-transcriptional regulators
Source: Nat Commun. 2023 Jan 11;14:166. doi: 10.1038/s41467-023-35853-1 (PMC9834408; doi:10.1038/s41467-023-35853-1)
Supplement: Supplementary file 10 — Reporting Summary [file 41467_2023_35853_MOESM10_ESM.pdf]

nature portfolio

Corresponding author(s): Dea Slade

Last updated by author(s): Dec 22, 2022

Reporting Summary

Nature Portfolio wishes to improve the reproducibility of the work that we publish. This form provides structure for consistency and transparency in reporting. For further information on Nature Portfolio policies, see our Editorial Policies and the Editorial Policy Checklist.

Statistics

For all statistical analyses, confirm that the following items are present in the figure legend, table legend, main text, or Methods section.

n/a	Confirmed	
☐	☒	The exact sample size (*n*) for each experimental group/condition, given as a discrete number and unit of measurement
☐	☒	A statement on whether measurements were taken from distinct samples or whether the same sample was measured repeatedly
☐	☒	The statistical test(s) used AND whether they are one- or two-sided<br>*Only common tests should be described solely by name; describe more complex techniques in the Methods section.*
☒	☐	A description of all covariates tested
☒	☐	A description of any assumptions or corrections, such as tests of normality and adjustment for multiple comparisons
☐	☒	A full description of the statistical parameters including central tendency (e.g. means) or other basic estimates (e.g. regression coefficient) AND variation (e.g. standard deviation) or associated estimates of uncertainty (e.g. confidence intervals)
☐	☒	For null hypothesis testing, the test statistic (e.g. *F*, *t*, *r*) with confidence intervals, effect sizes, degrees of freedom and *P* value noted<br>*Give P values as exact values whenever suitable.*
☒	☐	For Bayesian analysis, information on the choice of priors and Markov chain Monte Carlo settings
☒	☐	For hierarchical and complex designs, identification of the appropriate level for tests and full reporting of outcomes
☒	☐	Estimates of effect sizes (e.g. Cohen's *d*, Pearson's *r*), indicating how they were calculated

Our web collection on statistics for biologists contains articles on many of the points above.

Software and code

Policy information about availability of computer code

Data collection	X-ray diffraction datasets were collected at the beamline MASSIF ID30a1 (ESRF, Grenoble) at 100K using a wavelength of 0.966 Å (SHARP SPOC:1xS5P CTD and the beamline I04 (DLS, Didcot Oxfordshire) at 100K using a wavelength of 0.9795 Å (RBM15 SPOC).<br>Fluorescence Anisotropy data were recorded on a Perkin Elmer LS50 B fluorescence spectrometer using Perkin Elmer FL WinLab software (version 3.00).<br>SEC-MALLS data were collected on an Agilent Technologies 1260 Infinity HPLC equipped with a miniDawn Treos detector (Wyatt Technology) using a laser emitting at 690 nm. The HPLC was operated with OpenLAB CDS software (Agilent Technologies, Rev C.01.07 SR3 [465]), MALLS data acquisition was performed with ASTRA software (Wyatt Technology, version 7.3.2.19).<br>Mass Spectrometry data from Co-IP experiments were collected on a Q Exactive HF-X Orbitrap (Thermo Fisher; GFP-IPs) or an Orbitrap Fusion Lumos mass spectrometer (Thermo Fisher; FLAG-IPs).<br>Mass spectrometry data collection for analysis of m6A abundance were collected on an Agilent 1290 Infinity II equipped with a Phenomenex Synergi 2.5 µm Fusion-RP 100 Å (100 × 2 mm) coupled to an Agilent 6470 Triple Quad equipped with an ESI ion source.<br>Western Blots were imaged on a ChemiDoc MP Imaging system (Bio-Rad) operated by Bio-Rad Image Lab Touch Software (version 2.3.0.07)<br>Next generation sequencing was performed on a NextSeq 550 sequencer (Illumina) in readmode SR75 or a NovaSeq 600 sequencer (Illumina) in readmode SR100 or PE150.<br>Immunofluorescence images were acquired using an inverse point scanning confocal Zeiss LSM980 Microscope equipped with a Zeiss Plan-Apochromat 63x/1.4 Oil DIC M27 (WD 0.19 mm) running with Zeiss ZEN Blue 3.3 software (version 3.3.89.0008).<br>qPCR data were acquired using a C1000 Touch CFX384 Real-Time System (Bio-Rad) operated by Bio-Rad CFX Maestro software (version 2.2).<br>FACS during cell line generation was performed on a BD FACS Melody instrument operated by BD FACSChorus software (version 1.1.20.0).

Data analysis	X-ray data frames were processed using the XDS package (version January 26, 2018), and converted to mtz format with the program AIMLESS (version 0.7.7) with the help of autoprocessing pipelines at the synchrotrons. The structures were determined using the molecular replacement program PHASER (version 2.8.3). Structures were refined with Phenix Refine (version 1.20.1-4487) and rebuilt using Coot (version 0.9.654). Structural depictions were generated using UCSF Chimera (version 1.14).<br>Models of SPOC domain-peptide complexes were generated in Pymol (version 2.5.2) and refined using the HADDOCK 2.2 webserver.<br>Fluorescence Anisotropy data were analysed using QtiPlot 1.0.0-rc13 (version 5.9.8).<br>MALLS data analysis was performed with ASTRA software (Wyatt Technology, version 7.3.2.19).<br>Mass spectrometry data from Co-IP experiments were processed using the MaxQuant software package version 1.6.16.0 respect. 1.6.14.0. MaxQuant output tables were further processed in R (version 4.0.2). Statistical analysis was performed using the LIMMA package (version 3.54.0) at 5% FDR (Benjamini-Hochberg).<br>Quantification of nucleosides was done using calibration curves of synthetic standards and stable isotope labeled internal standards using Agilent's MassHunter software (version 9.0.647.0).<br>Western Blots were analysed using Bio-Rad Image Lab Software (version 5.2.1).<br>RNA-seq data were processed using PiGx-RNA-seq pipeline (version 0.1.0, DOI: 10.1093/gigascience/giy123). Data were quantified using the GRCh38/hg38, and the dm6 versions of the human and Drosophila spike-in transcriptome (downloaded from the ENSEMBL database, doi: 10.1093/nar/gkx1098) using SALMON (version 1.9.0, DOI: 10.1038/nmeth.4197) with default parameters. For visualization purposes, the data was mapped to the GRCh38/hg38, and dm6 versions of the human, and drosophila genomes using STAR (version 2.7.10a), with the following parameters: --limitOutSJcollapsed 20000000 --limitIObufferSize=1500000000 --outFilterMultimapNmax 10 --seedPerWindowNmax 5. The quantified data was processed using tximport (26925227), and the differential expression analysis was done using DESeq2 (version 1.38.1, DOI: 10.1186/s13059-014-0550-8).<br>TT-seq data were processed using PiGx-RNA-seq pipeline (version 0.1.0, DOI: 10.1093/gigascience/giy123). Data was quantified using the GRCh38/hg38, and the dm6 versions of the human, and labeled and unlabeled spike-in sequences (downloaded from the ENSEMBL database doi: 10.1093/nar/gkx1098) using SALMON (version 1.9.0, DOI: 10.1038/nmeth.4197) with default parameters. The data was mapped to the GRCh38/hg38 version of the human genome using STAR (version 2.7.10a), with the following parameters: --limitOutSJcollapsed 20000000 --limitIObufferSize=1500000000. The STAR genome index was created using the following parameter: --genomeSuffixLengthMax 300. The differential initiation and stalling index were calculated using DESeq2 (version 1.38.1, DOI: 10.1186/s13059-014-0550-8).<br>ChIP-seq data were processed using the PiGX - ChIPSeq pipeline (version 0.1.0). Data were mapped to the hg38 version of the human genome using Bowtie2 (version 2.4.5) with the following parameters: bowtie2 -k 1. The data was normalized using DESeq2 derived size factors. Differentially bound regions were defined using DESeq2 (version 1.38.1, DOI: 10.1186/s13059-014-0550-8).<br>The Airyscan images were processed for super-resolution with Zen Blue 3.3 (version 3.3.89.0008) Auto Airyscan filter and furthermore thresholded using Fiji/ImageJ software (version 2.1.0/1.52c) with Costes-related automatic thresholds for each channel in each experiment for better digital and analog display.<br>qPCR data were analysed and plotted using Microsoft Excel 365 (version 16.58) and GraphPad Prism 9 (version 9.2.0).

For manuscripts utilizing custom algorithms or software that are central to the research but not yet described in published literature, software must be made available to editors and reviewers. We strongly encourage code deposition in a community repository (e.g. GitHub). See the Nature Portfolio guidelines for submitting code & software for further information.

Data

Policy information about availability of data

All manuscripts must include a data availability statement. This statement should provide the following information, where applicable:

- Accession codes, unique identifiers, or web links for publicly available datasets
- A description of any restrictions on data availability
- For clinical datasets or third party data, please ensure that the statement adheres to our policy

The source data are provided with this paper. The atomic coordinates have been deposited in the Protein Data Bank under accession codes: 7Z27 (https://doi.org/10.2210/pdb7Z27/pdb) for RBM15 SPOC and 7Z1K (https://doi.org/10.2210/pdb7Z1K/pdb) for SHARP SPOC:1xS5P CTD. The sequencing data generated in this study have been deposited in ArrayExpress under accession code: E-MTAB-12358 (https://www.ebi.ac.uk/biostudies/arrayexpress/studies/E-MTAB-12358?accession=E-MTAB-12358) (RNA-seq), E-MTAB-12359 (https://www.ebi.ac.uk/biostudies/arrayexpress/studies/E-MTAB-12359?accession=E-MTAB-12359) (TTchem-seq), E-MTAB-11506 (https://www.ebi.ac.uk/biostudies/arrayexpress/studies/E-MTAB-11506?accession=E-MTAB-11506) (ChIP-seq). The processed sequencing data are provided in Supplementary Data 3, 4 and 5. Mass spectrometry data have been deposited to the jPOST repository66 under the accession number JPST001505 (https://repository.jpostdb.org/entry/JPST001505) (anti-FLAG IP) and JPST001502 (https://repository.jpostdb.org/entry/JPST001502) (anti-GFP IP). The processed mass spectrometry data are provided in Supplementary Data 1 and 2. Atomic coordinates used in this study are available in the Protein Data Bank under accession codes 2RT5, 1OW1, 6QV2, 6IC8 and 5KXF and in the Alpha Fold Protein Structure Database under accession codes Q9BTC0, Q6ZMY3 and Q96T37.

Human research participants

Policy information about studies involving human research participants and Sex and Gender in Research.

Reporting on sex and gender	The study did not involve any human research participants.
Population characteristics	Not applicable.
Recruitment	Not applicable.
Ethics oversight	Not applicable.

Note that full information on the approval of the study protocol must also be provided in the manuscript.

Field-specific reporting

Please select the one below that is the best fit for your research. If you are not sure, read the appropriate sections before making your selection.

- [x] Life sciences
- [ ] Behavioural & social sciences
- [ ] Ecological, evolutionary & environmental sciences

For a reference copy of the document with all sections, see nature.com/documents/nr-reporting-summary-flat.pdf

Life sciences study design

All studies must disclose on these points even when the disclosure is negative.

Sample size

No statistical methods were used to predetermine sample size. Sample sizes were chosen based on our previous experience and similar studies. Fluorescence anisotropy assays, Co-IPs followed by mass spectrometry and nucleoside mass spectrometry were performed in triplicates because this allows for statistical analysis and detection and removal of potential outliers. Since we were in all cases able to replicate our results in all three replicates, we deemed the sample size sufficient.

Co-IPs followed by Western Blotting were performed once, since they confirmed the results of mass spectrometry analysis.

Sample sizes and sequencing depth for NGS experiments were chosen based on the standards recommended by the ENCODE Consortium and on previously published similar studies. ENCODE recommends two or more biological replicates for RNA-seq and ChIP-seq. We chose to perform RNA-seq, TT-seq and PHF3-GFP ChIP-seq in three biological replicates because this allows for detection and removal of potential outliers. We performed SHARP-GFP ChIP-seq in two biological replicates since only one region of enriched genomic binding was detected in the first replicate, which was confirmed in the second replicate. All attempts at replication of NGS data were successful with the exception of two TT-seq samples which had to be excluded for technical reasons (see Data exclusions), therefore we consider the sample size sufficient.

ChIP-seq results were additionally confirmed by ChIP-qPCR, which was performed in four replicates to allow for reliable detection of potential outliers and calculation of statistical significance.

Immunofluorescence analysis was performed in biological duplicates and multiple cells per replicate were imaged. Since replication was successful, we consider the sample size sufficient.

Genotyping PCRs and Western Blots during cell line generation were only performed once, since they were meant to confirm genotype and protein expression and not to generate experimental data.

Data exclusions

PHF3_dSPOC_br3 and DIDO_dSPOC_br3 were removed from the TT-seq analysis, because they had an extreme genome to spike in ratio.

Replication

All attempts at replication were successful. Fluorescence anisotropy, mass spectrometry, RNA-seq and TT-seq were performed in triplicates. Individual replicates from two TT-seq samples were excluded for technical reasons (see Data exclusions), but the remaining two replciates for these samples showed successfull replication. ChIP-seq was performed in triplicates (PHF3) or duplicates (SHARP). ChIP-qPCR was performed in four replicates. Immunofluorescence was performed in biological duplicates and replicated successfully.

Co-IPs followed by Western Blotting were perfomed once, but confirmed the findings from Co-IP mass spectrometry analysis.

Structural studies/X-ray crystallography were not replicated, but the solved structures show very high similarity to previously published structures of SHARP SPOC (PDB 2RT5) or a structural model of RBM15 SPOC generated by AlphaFold2 (Q96T37).

Experiments meant to confirm successful CRISPR/Cas9 editing rather than generate experimental data (genotyping PCRs, Western Blots) were not replicated.

Randomization

Randomization was not performed for this study. Samples (cell lines) were allocated to experimental groups based on genotype and paired with a control, i.e. KO and ΔSPOC cell lines were compared to a control WT cell line or FLAG-SPOC domain transfected cells were compared to empty vector transfected control cells. All cell lines were grown under the same conditions and experiments were performed under identical conditions.

Blinding

The investigators were not blinded in this study since they were involved in the planning, execution and analysis of the experiments. Within-experiment sample groups and the respective controls were prepared, processed and analysed at the same time and under identical conditions to eliminate any bias, so prior knowledge had no impact on data output. No subjective process was involved in the analysis of the data. Analysis of NGS- and mass spectrometry data was carried out bioinformatically applying the same parameters to all samples without need for investigator blinding.

Reporting for specific materials, systems and methods

We require information from authors about some types of materials, experimental systems and methods used in many studies. Here, indicate whether each material, system or method listed is relevant to your study. If you are not sure if a list item applies to your research, read the appropriate section before selecting a response.

Materials & experimental systems

n/a	Involved in the study
[ ]	[x] Antibodies
[ ]	[x] Eukaryotic cell lines
[x]	[ ] Palaeontology and archaeology
[x]	[ ] Animals and other organisms
[x]	[ ] Clinical data
[x]	[ ] Dual use research of concern

Methods

n/a	Involved in the study
[ ]	[x] ChIP-seq
[ ]	[x] Flow cytometry
[x]	[ ] MRI-based neuroimaging

Antibodies

Antibodies used

Rabbit Anti-GFP (polyclonal), Abcam ab290
Mouse anti-FLAG clone M2-peroxidase, Sigma A8592
Mouse anti-Pol II clone F-12, Santa Cruz 55492
Rabbit anti-SPT6 (polyclonal), Novus Biologicals NB100-2582
Mouse anti-DSIF clone 17, Becton Dickinson 611107
Rabbit anti-PAF1 (polyclonal), Abcam ab20662
Rabbit anti-Leo1 (polyclonal), Bethyl A300-174A
Rabbit anti-CK2α (polyclonal), Cell Signaling 2656
Rat anti-ZNF768 clone 5c8, provided by Dirk Eick
Rabbit anti-MSH2 clone D24B5, Cell Signaling 2017
Mouse anti-HTATSF1 clone C-4, Santa Cruz 514351
Rabbit anti-RBM15 (polyclonal), Bethyl A300-821A
Rabbit anti-PCNA (polyclonal), Abcam ab18197
Rabbit anti-WTAP clone E7O5B, Cell Signaling 56501
Rabbit anti-CHK2 (polyclonal), Cell Signaling 2662
Rabbit anti-IWS1 (polyclonal), Cell Signaling 5681
Mouse anti-FMR1 clone 1F1, Merck Millipore MABN2453
Rabbit anti-DIDO1 (polyclonal), Atlas antibodies HPA049904
Mouse anti-α-tubulin clone B-5-1-2, Sigma, T6074
Goat anti-mouse Alexa Fluor 568 (polyclonal), Invitrogen A11004
Goat anti-rabbit Alexa Fluor 568 (polyclonal), Invitrogen A11011

Validation

Antibodies were validated by the manufacturers or in previously published studies, used extensively in literature, or validated in this study.
Rabbit anti-GFP (Abcam ab290): Manufacturer's statement: Anti-GFP antibody (ab290) is a highly versatile antibody that gives a stronger signal than other anti-GFP antibodies available. On Western blot the antibody detects the GFP fraction from cell extracts expressing recombinant GFP fusion proteins and has also been shown to be useful on mouse sections fixed with formalin. In Immunocytochemistry, the antibody gives a very good signal on recombinant YES-GFP chimeras expressed in COS cells (McCabe et al. 1999 and figure below). It is routinely used in Immunoprecipitation (IP) and IP-Western protocols and has been used successfully in HRP Immunohistochemistry at 1:200 on whole-mount mouse embryos.
Mouse anti-FLAG M2-peroxidase (Sigma A8592): used in >50 publications and validated in this study (Fig. 5a-e, empty vector vs. FLAG-tagged constructs).
Mouse anti-Pol II clone F-12 (Santa Cruz sc-55492): used in >30 publications and validated in our previous study (https://doi.org/10.1038/s41467-021-26360-2) using purified Pol II complex in WB and in ChIP.
Rabbit anti-SPT6 (Novus Biologicals NB100-2582): Validated by the manufacturer using siRNA knockdown to confirm the specificity of Spt6 antibody in C2C12 myoblasts. The antibody was validated for Western Blotting in human cells in Lu et al. 2020 (https://doi.org/10.1172/JCI138577), where it was used to confirm SPT6 depletion in MDA-MB-231 cells transfected with shRNAs targeting SPT6 by Western Blotting.
Mouse anti-DSIF (Becton Dickinson 611107) - Manufacturer's statement: 'This antibody is routinely tested by western blot analysis.'
Rabbit anti-PAF1 (Abcam ab20662) - Manufacturer's statement (WB): 'ab20662 recognizes a band at approximately 80kDa, which corresponds in size to PAF1 / PD2. Although it has a calculated molecular weight of ~60kDa, several groups have shown that it migrates at a size of 80-90kDa (see Moniaux, et.al., Oncogene Feb. 2006, Yart et.al., Mol Cell Biol 25:5052-60, 2005, and Rozenblatt-Rosen, et.al., Mol Cell Biol 25:612-20, 2005). There is also a smaller band at ~73kDa which may be a degradation product. Both bands are competed away by the addition of immunizing peptide, suggesting that the interaction is specific.'
Rabbit anti-Leo1 (Bethyl A300-174A): Validated by the manufacturer for detection of human and mouse Leo1 by western blot. Samples: Whole cell lysate (50 µg) from HeLa, HEK293T,
Jurkat, mouse TCMK-1, and mouse NIH 3T3 cells prepared using NETN lysis buffer.Validated in Karmakar et al., 2020 (https://doi.org/10.1053/j.gastro.2020.07.053) for Western Blotting in human cell lines. The antibody was used to confirm LEO1 knock-down in SW1990 cells after transfection with Scramble siRNA or LEO1 siRNA, confirming the specificity of the antibody.
Rabbit anti-CK2α (Cell Signaling 2656): Used in >30 publications. Statement from the manufacturer: This antibody detects endogenous levels of total CK2α 1 protein. This antibody may cross-react with CK2α prime. An image of Western Blot detection of CK2α in lysates from A673, HT1376, HeLa and NIH/3T3 cell lines is provided on the manufacturer's website. This antibody is further validated for Western Blotting in human cells in Hussein et al. 2021 (https://doi.org/10.3390/cells10071770) where it is used to confirm knock-down of CK2α in U2OS and KHOS/NP cells.
Rat anti-ZNF768: provided by Dirk Eick, validated in doi: 10.1093/nar/gky1148

Rabbit anti-MSH2 (Cell signaling #2017): Statement from the manufacturer on specificity: MSH2 (D24B5) XP® Rabbit mAb detects endogenous levels of total MSH2 protein. An image of Western Blot detection of MSH2 in lysates from HeLa and NIH/3T3 cells in provided on the manufacturer's website. The antibody was further validated for Western blotting in human cell lines in Zhu et al., 2022 (https://doi.org/10.3389/fonc.2022.799475) where it was used to detect MSH2 levels in BxPC-3 cells.
Mouse anti-HTATSF1 (Santa Cruz sc-514351): specific for an epitope mapping between amino acids 1-23 at the N-terminus of HIV-1 Tat-SF1 of mouse origin. Tested by the manufacturer in Western Blotting using non-transfected 293T cells and 293T cells transcfected with HIV-1 Tat-SF1, as well as whole cell lysates from various cell lines.
Rabbit anti-RBM15 (Bethyl A300-821A): Tested by the manufacturer in Western Blotting using several cell lines and in IP. Images for Western Blot detection of RBM15 in human cell lines HeLa, HEK 293T, Jurkat, K-562, RKO and MCF-7 are provided on the manufacturer's website. Validated in this study, where it was used to detect loss of RBM15 protein after CRISPR/Cas9 knock-out and size shift of RBM15 protein after deletion of the SPOC domain by Western Blotting. The antibody was not previously validated for IF.
Rabbit anti-PCNA (Abcam ab18197): Images of Western blot detection of PCNA in whole cell lysate from HEK293 cells and of purified recombinant human PCNA protein are provided on the manufacturer's website. The antibody is used in >50 publications, Western Blot detection of PCNA in human cells is e.g. shown in Kim et al., 2020 (10.1093/nar/gkaa501), Cooper et al., 2015 (10.1080/15384101.2015.1053667), Gilljam et al., 2012 (https://doi.org/10.1371/journal.pone.0049199) and Ciccia et al, 2012 (10.1016/j.molcel.2012.05.024).
Rabbit anti-WTAP (Cell Signaling 56501): Manufacturer's statement on specificity: WTAP Antibody recognizes endogenous levels of total WTAP protein. Tested in Western Blotting using several cell lines.
Rabbit anti-Chk2 (Cell Signaling 2662): Manufacturer's statement on specificity: Chk2 Antibody detects endogenous levels of total Chk2 protein independent of phosphorylation. Images of Western Blot detection in human cell lines GM536 and M059J are provided on the manufacturer's website. The antibody is used in >200 publications. Western Blot detection of CHK2 in human cells is shown in Verdun et al., 2005 (https://doi.org/10.1016/j.molcel.2005.09.024), Qin et al., 2019 (10.1038/s41467-019-09175-0), Leimbacher et al. 2019 (10.1016/j.molcel.2019.02.014), Göder et al., 2018 (10.1038/s41467-018-03096-0) and further studies.
Rabbit anti-IWS1 (Cell Signaling 5681):Manufacturer's statement on specificity: IWS1 Antibody detects endogenous levels of total IWS1 protein. Tested by Western blotting using several cell lines.
Mouse anti-FMR1 (Merck Millipore MABN2453): Manufacturer's statement on specificity: Clone 1F1 is a mouse monoclonal antibody that specifically detects Fragile X mental retardation protein 1 homolog (FMR1). It targets an epitope with in the C-terminal half. It has been tested in Western Blotting.
Rabbit anti-DIDO1 (Atlas antibodies HPA049904): Manufacturer's statement on validation: Validated in Western blot using relevant lysates. An image of Western Blot detection in the human cell line RT-4 using this antibody is shown on the manufacturer's website. Target specificity was validated in this study, where it was used to detect loss of DIDO protein after CRISPR/Cas9 knock-out and size shift of DIDO protein after deletion of the SPOC domain by Western Blotting.
Mouse anti-α-tubulin (Sigma T6074): The antibody's target specificity was validated by the manufacturer's 'Antibody Enhanced Validation - Independent Antibody Verification' procedure. Manufacturer's statement on the procedure: Demonstrating antibody specificity through the use of multiple antibodies against target in IHC or ICC. Expected results: All antibodies should show similar staining patterns or experimental results. The validity of results obtained with an antibody in a given immunoapplication may be supported by showing that the same results are obtained using the identical protocol with a different antibody raised against the same target. At least two antibodies with non-overlapping epitopes are applied across a panel of samples, such as sections from the same tissue. This approach has the added advantage of enabling validation of both antibodies used for comparison of binding characteristics. Images displaying Western blot detection of α-tubulin in lysate from human cell lines (HeLa, Jurkat) are shown on the manufacturer's website and the antibody was used in >100 publications.
Goat anti-mouse Alexa Fluor 568 (Invitrogen A11004): Manufacturer's statement on antibody testing in IF in human cells: Immunofluorescence analysis of Goat anti-Mouse IgG (H+L) Cross-Adsorbed Secondary Antibody, Alexa Fluor® 568 conjugate was performed using HeLa cells stained with alpha Tubulin (236-10501) Mouse Monoclonal Antibody (Product # A11126). The cells were fixed with 4% paraformaldehyde for 10 minutes, permeabilized with 0.1% Triton™ X-100 for 10 minutes, blocked with 1% BSA for 1 hour and labeled with 2 µg/mL primary antibody for 3 hours at room temperature. Goat anti-Mouse IgG (H+L) Cross-Adsorbed Secondary Antibody, Alexa Fluor® 568 (Product # A-11004) was used at a concentration of 2 µg/mL in phosphate buffered saline containing 0.2% BSA for 45 minutes at room temperature, for detection of alpha Tubulin in the cytoplasm (Panel a: red). Nuclei (Panel b: blue) were stained with DAPI in SlowFade® Gold Antifade Mountant (Product # S36938). F-actin was stained with Alexa Fluor® 488 Phalloidin (Product # A12379), 1:300) (Panel c: green). Panel d represents the composite image. No nonspecific staining was observed with the secondary antibody alone (panel f), or with an isotype control (panel e). The images were captured at 60X magnification.
Goat anti-rabbit Alexa Fluor 568 (Invitrogen A11011): Manufacturer's statement on antibody testing in IF in human cells: Immunofluorescence analysis of Goat anti-Rabbit IgG (H+L) Cross-Adsorbed Secondary Antibody Alexa Fluor® 568 conjugate was performed using HeLa cells stained with alpha Tubulin Rabbit Polyclonal Antibody (Product # PA5-16891) The cells were fixed with 4% paraformaldehyde for 10 minutes, permeabilized with 0.1% Triton™ X-100 for 10 minutes, blocked with 1% BSA for 1 hour and labeled with 2 µg/mL primary antibody for 3 hours at room temperature. Goat anti-Rabbit IgG (H+L) Cross-Adsorbed Secondary Antibody Alexa Fluor® 568 conjugate (Product # A-11011) was used at a concentration of 2 µg/mL in phosphate buffered saline containing 0.2% BSA for 45 minutes at room temperature, for detection of alpha Tubulin in the cytoplasm (Panel a: red). Nuclei (Panel b: blue) were stained with DAPI in SlowFade® Gold Antifade Mountant (Product # S36938). F-actin was stained with Alexa Fluor® 488 Phalloidin (Product # A12379), 1:300) (Panel c: green). Panel d represents the composite image. No nonspecific staining was observed with the secondary antibody alone (panel f), or with an isotype control (panel e). The images were captured at 60X magnification.

Eukaryotic cell lines

Policy information about cell lines and Sex and Gender in Research

Cell line source(s)	HEK 293T cells and Drosophila S2 cells were acquired from ATCC.
Authentication	HEK 293T and Drosophila S2 cells were not authenticated.

Mycoplasma contamination

All cell lines used in the study were tested for mycoplasma contamination by PCR and tested negative.

Commonly misidentified lines
(See ICLAC register)

No commonly misidentified cell lines were used in this study.

ChIP-seq

Data deposition

☒ Confirm that both raw and final processed data have been deposited in a public database such as GEO.

☒ Confirm that you have deposited or provided access to graph files (e.g. BED files) for the called peaks.

Data access links
May remain private before publication.

The sequencing data generated in this study have been deposited in ArrayExpress under accession code: E-MTAB-11506 [https://www.ebi.ac.uk/arrayexpress/experiments/E-MTAB-11506/]

Files in database submission

ChIP_HEK_dSPOC_PHF3-GFP_br1.fastq.gz
ChIP_HEK_dSPOC_PHF3-GFP_br2.fastq.gz
ChIP_HEK_dSPOC_PHF3-GFP_br3.fastq.gz
ChIP_HEK_dSPOC_SHARP-GFP_br1.fastq.gz
ChIP_HEK_dSPOC_SHARP-GFP_br2.fastq.gz
ChIP_HEK_WT_PHF3_br1.fastq.gz
ChIP_HEK_WT_PHF3_br2.fastq.gz
ChIP_HEK_WT_PHF3_br3.fastq.gz
ChIP_HEK_WT_PHF3-GFP_br1.fastq.gz
ChIP_HEK_WT_PHF3-GFP_br2.fastq.gz
ChIP_HEK_WT_PHF3-GFP_br3.fastq.gz
ChIP_HEK_WT_SHARP-GFP_br1.fastq.gz
ChIP_HEK_WT_SHARP-GFP_br2.fastq.gz

Genome browser session
(e.g. UCSC)

https://bimsbstatic.mdc-berlin.de/hubs/akalin/SPOC/hub.txt

Methodology

Replicates

3 replicate of PHF3 ChIP-seq and 2 replicates of SHARP ChIP-seq were performed and validated with a further 4 replicates of ChIP-qPCR.

Sequencing depth

ChIP_HEK_dSPOC_PHF3-GFP_br1 33159104
ChIP_HEK_dSPOC_PHF3-GFP_br2 34653879
ChIP_HEK_dSPOC_PHF3-GFP_br3 33998605
ChIP_HEK_dSPOC_SHARP-GFP_br1 42280867
ChIP_HEK_dSPOC_SHARP-GFP_br2 49175099
ChIP_HEK_WT_PHF3_br1 32959346
ChIP_HEK_WT_PHF3_br2 39974118
ChIP_HEK_WT_PHF3_br3 25019941
ChIP_HEK_WT_PHF3-GFP_br1 42282041
ChIP_HEK_WT_PHF3-GFP_br2 32587883
ChIP_HEK_WT_PHF3-GFP_br3 30067450
ChIP_HEK_WT_SHARP-GFP_br1 43537257
ChIP_HEK_WT_SHARP-GFP_br2 46313006

Antibodies

GFP (Abcam ab290)

Peak calling parameters

Not applicable, peak calling was not performed for analyis of ChIP data because the signal was enriched over large genomic areas rather than distinct peaks.

Data quality

Sequencing quality control was performed using FastQC. Data quality estimation was based on mapping statistics and visual inspection in IGV browser.

Software

ChIP seq data was processed using the PiGX - ChIPSeq pipeline. The data was mapped to the hg38 version of the human genome using Bowtie2 with the k = 1 parameter. Data was normalized using DESeq2 derived size factors. Differentially bound regions were defined using DESeq2

Flow Cytometry

Plots

Confirm that:

- [x] The axis labels state the marker and fluorochrome used (e.g. CD4-FITC).
- [x] The axis scales are clearly visible. Include numbers along axes only for bottom left plot of group (a 'group' is an analysis of identical markers).
- [ ] All plots are contour plots with outliers or pseudocolor plots.
- [ ] A numerical value for number of cells or percentage (with statistics) is provided.

Methodology

Sample preparation	Cells were trypsinized and centrifuged for 5min at 500g. The cell pellet was resuspended in FACS medium (DMEM+Gln+50% FBS) and passed into FACS tubes through the sieve in the cap.
Instrument	FACS sortings were performed on a FACSMelody cell sorter (BD), serial number R6627540033.
Software	The cell sorter was operated by BD FACSChorus software (version 1.1.20.0).
Cell population abundance	FACS was used for cell sorting during cell line generation for the following purposes:<br>- Sorting of GFP-positive cells 48-72h after transfection with pX458 Cas9-EGFP plasmids to enrich for transfected cells. The abundance of the sorted population (GFP+ high) was typically between 10% and 80% of the total population depending on transfection efficiency.<br>- Sorting of GFP-negative cells 1-2 weeks after transfection with pX458 Cas9-EGFP plasmids to exclude cells with persistent Cas9-EGFP expression. The abundance of the sorted population (GFP-) was typically >90% of the total population.<br>- Sorting of GFP-positive cells during endogenous GFP-tagging to enrich for tagged cells. The abundance of the sorted population was typically 0.5% to 10% of the total population depending on the editing efficiency and whether previous enrichment steps (antibiotic selection) had been conducted.<br>Purity of the post-sort fractions was not assessed because single cell sorting was performed and clonal cell populations were validated on the genomic (genotyping PCR) and protein level (Western Blot) before proceeding with experiments.
Gating strategy	The gating strategy is illustrated in Supplementary Fig. 15. Singlet population was defined by forward versus side scatter (FSC vs. SSC) gating. Within the singlet population, three populations were gated based on their FITC/GFP fluorescence: GFP- (negative), GFP+ low (endogenous GFP expression) and GFP+ high (exogenous GFP expression). Histograms depict the distribution of cells over the GFP-groups.

- [x] Tick this box to confirm that a figure exemplifying the gating strategy is provided in the Supplementary Information.
